# An Insight Into Pentatricopeptide-Mediated Chloroplast Necrosis *via* microRNA395a During *Rhizoctonia solani* Infection

**DOI:** 10.3389/fgene.2022.869465

**Published:** 2022-05-30

**Authors:** Nagesh Srikakulam, Ashirbad Guria, Jeyalakshmi Karanthamalai, Vidya Murugesan, Vignesh Krishnan, Kasthuri Sundaramoorthy, Shakkhar Saha, Rudransh Singh, Thiveyarajan Victorathisayam, Veeraputhiran Rajapriya, Ganapathi Sridevi, Gopal Pandi

**Affiliations:** Department of Plant Biotechnology, School of Biotechnology, Madurai Kamaraj University, Madurai, India

**Keywords:** miRNA, Small RNA sequencing, *R. solani*, precursor miRNA, pentatricopeptide, chloroplast necrosis, rice

## Abstract

Sheath blight (ShB) disease, caused by *Rhizoctonia solani,* is one of the major biotic stress-oriented diseases that adversely affect the rice productivity worldwide. However, the regulatory mechanisms are not understood yet comprehensively. In the current study, we had investigated the potential roles of miRNAs in economically important indica rice variety Pusa Basmati-1 upon *R. solani* infection by carrying out in-depth, high-throughput small RNA sequencing with a total data size of 435 million paired-end raw reads from rice leaf RNA samples collected at different time points. Detailed data analysis revealed a total of 468 known mature miRNAs and 747 putative novel miRNAs across all the libraries. Target prediction and Gene Ontology functional analysis of these miRNAs were found to be unraveling various cellular, molecular, and biological functions by targeting various plant defense-related genes. Quantitative reverse transcription polymerase chain reaction (qRT-PCR) was performed to validate the miRNAs and their putative target genes. Out of the selected miRNA-specific putative target genes, miR395a binding and its cleavage site on pentatricopeptide were determined by 5’ RACE-PCR. It might be possible that *R. solani* instigated chloroplast degradation by modulating the pentatricopeptide which led to increased susceptibility to fungal infection.

## Introduction

Rice is the most economically important staple food crop which is preferred over other food crops in India that provides a vital part of the daily dietary intake and nutritional needs of ∼70% of the world’s population [Food and Agriculture Organization (FAO), 1995]. Therefore, the production of rice is very important for global food security. According to FAO, the world rice production is 757 million tonnes/164 million hectares (MT/Mha) in 2020 compared to 538 MT/147 Mha in 1994 suggesting an increasing trend of rice production in hectograms per hectare (hg/ha) land usage in 2020 (46,618 hg/ha) as compared to 1994 (36,576 hg/ha) (https://www.fao.org/faostat/en/#data/QCL/visualize). In spite of such importance, the global production of rice is greatly impacted by several environmental factors such as diverse biotic and abiotic stresses. Rice diseases are caused by diverse pathogens including fungi, bacteria, viruses, and nematodes (Ling, 1980). Among microbial diseases in plants, fungi are responsible for 70–80% of crop losses amounting to 125 MT (Fisher et al., 2012; Godfray et al., 2016; https://www.imperial.ac.uk/news/108986/tackle-fungal-forces-save-crops-forests/). This scenario could further aggravate in the future with the tentative increase in the fungal infection rate due to the changes in climatic conditions.

*Rhizoctonia solani* Kühn AG1-IA (anamorph) [*Thanatephorus cucumeris* (Frank) Donk (teleomorph)] is a soil-born plant pathogenic fungus that causes sheath blight (ShB) disease in rice. ShB is alternatively named as “oriental leaf and sheath blight,” “brown-bordered leaf and sheath spot,” and “snake skin” (Lee and Rush, 1983; Molla et al., 2020). It is the most severe fungal disease in rice worldwide next to *Magnaporthe grisea* (which causes rice blast) that led to a devastating loss in rice productivity and grain quality (Marchetti and Bollich, 1991). *R. solani* is a necrotrophic pathogen that persists in the soil in the form of spores, and it survives either as sclerotia or mycelia in the host plants. The sclerotia float on the water in the rice fields, probably reaching the sheath and stem, thereby causing infection (Ou, 1985). *R. solani* infection causes up to a 20–50% decrease in the rice yield under moist and wet environmental conditions (Lee and Rush, 1983; Marchetti and Bollich, 1991). In order to control *R. solani* infection, numerous studies were conducted such as over-expressing the pathogenesis-related (PR) protein and other defense-related genes which resulted in around 60–70% reduction in the infection (Sridevi et al., 2003, 2008; Shah et al., 2009, 2013; Sripriya et al., 2017). However, complete elimination could not be achieved which ultimately hamper the global rice production.

RNA interference (RNAi) is a gene regulatory phenomenon expressed at the DNA or transcript level that is mediated by small non-coding RNAs (ncRNAs) (Baulcombe, 2000; Matzke et al., 2001; Voinnet, 2009; Wilson and Doudna, 2013). MicroRNAs (miRNAs) are one such type of small ncRNAs, having 18–27 nucleotides (nt) length, that are well-known for regulation of gene expression, modification of chromosome structure, and protection from mobile elements (Baulcombe, 2000; Matzke et al., 2001; Voinnet, 2009; Wilson and Doudna, 2013). MiRNAs evolved as efficient defense regulators in plants to protect themselves from pathogen attacks (Baldrich et al., 2015; Baldrich and san Segundo, 2016). Similarly, several studies have been conducted on rice upon fungal infection to understand the role of miRNAs especially after *M. oryzae* and/or *R. solani* infection (Molla et al., 2020). However, most studies were concentrated on rice blast and less investigation was reported to be found on ShB disease in rice (Chopperla et al., 2020; Wenlei et al., 2020). Therefore, there is an urgent need toward discovering an alternative strategy to produce *R. solani*-resistant rice variety for its sustainable cultivation.

In the current study, a total of 24–613 novel and 211–392 known mature miRNAs were identified from 18 Illumina-sequenced RNA libraries extracted from indica rice, Pusa Basmati-1 (PB-1), collected at different time points post *R. solani* infection. Few selected miRNAs, including a novel miRNA (NmiR1), were validated by reverse transcription-polymerase chain reaction (RT-PCR) and stem-loop (SL)-RT-PCR, whereas their expression dynamics was analyzed by DESeq and quantitative (q)RT-PCR. Furthermore, Gene Ontology (GO) analysis identified a total of 10 miRNA target genes from five miRNAs whose time-dependent expression was understood by qRT-PCR. 5′ RNA ligase mediated-rapid amplification of cDNA ends (5′ RLM-RACE)-PCR was employed to find out the binding and cleavage site of miR395a-targeted pentatricopeptide (Os12t0109300-01) mRNA. These results suggested that *R. solani* tentatively stimulated chloroplast degradation by modulating the pentatricopeptide which is known to involve in the organellar RNA metabolism (Barkan et al., 1994; Fisk et al., 1999; Meierhoff et al., 2003; Okuda et al., 2007, 2009; O’Toole et al., 2008; Tang et al., 2010, 2017; Boussardon et al., 2012; Asano et al., 2013; Chateigner-Boutin et al., 2013; Hayes et al., 2013; Schallenberg-Rüdinger et al., 2013; Lin et al., 2015; Wagoner et al., 2015; Wu et al., 2016; Wang et al., 2017; Wang et al., 2018; Chen et al., 2019; Huang et al., 2020; Lv et al., 2020).

## Materials and Methods

### Plant Material

Initially, healthy PB-1 rice seeds were placed on the sterile wet blotting paper in Petri plates in the dark for 1 week at room temperature. The 1-week grown ∼50 seedlings were transferred to pots containing puddled soil and grown inside greenhouse at a constant temperature (∼32^⁰^C) and ∼80–85% humidity for 45 days. A total of ∼30 healthy rice plants of equal height containing 3–6 greenish tillers were selected for the *R. solani* infection process.

### *R. solani* Infection and Sample Collection

*R. solani* infection was performed according to Marshall and Rush (1980) and Sridevi et al. (2008). Briefly, *R. solani* cultures maintained on potato dextrose agar (PDA) were inoculated on the rice plants at the maximum tillering stage (45 days old in a greenhouse pot). The agar block containing mycelium culture was placed inside the tiller sheaths and wrapped around with moist cotton to provide a favorable environment for infection inside greenhouse, whereas the control plants were wrapped with wet cotton only without any *R. solani* inoculum. The leaf sheaths were collected at 0-hour post infection (hpi) (control), 24, 60, and 72 hpi in biological triplicate and at 12, 36, and 48 hpi in biological replicates. The infected plants were thereafter maintained in a greenhouse for visualizing symptom development.

### Small RNA Library Construction and Sequencing

Total RNA was isolated using RNAiso Plus as per the manufacturer’s instructions (Takara Bio, Kusatsu, Shiga, Japan). RNA of ∼10 μg was subjected to 2 U TURBO DNase (2 U/μl, Invitrogen, Thermo Scientific, Waltham, MA, United States) treatment by incubating at 37°C for 30 min followed by enzyme inactivation with 0.01 mM EDTA (Sigma, St. Louis, MO, United States) at 72°C for 10 min. Subsequently, the mixture was purified with phenol-chloroform and isopropanol precipitation. The DNase-treated RNA was tested for its integrity, purity, and quantification using Nanodrop ND-1000 (Thermo Scientific, Waltham, MA, United States), 0.8% agarose (SeaKem, LE Agarose, Lonza, United States) gel electrophoresis, and Bioanalyzer 2100 (Agilent, Santa Clara, CA, United States). Superior quality RNA having RNA integrity number (RIN) > 8 was used for small RNA (sRNA) library preparation using TruSeq Small RNA Library Preparation Kit (Illumina, San Diego, CA, United States) as per manufacturer’s instructions. The quality of the library was verified using a Qubit fluorometer (Thermo Scientific, Waltham, MA, United States) and Bioanalyzer 2100. The qualified libraries were used for sequencing on the HiSeq 2500 platform (Illumina, San Diego, CA, United States) for obtaining estimated 25 million paired-end reads per library (outsourced to SciGenom Pvt. Ltd., Kochi, Kerala, India).

### MiRNA Analysis

**FIGURE 1:**
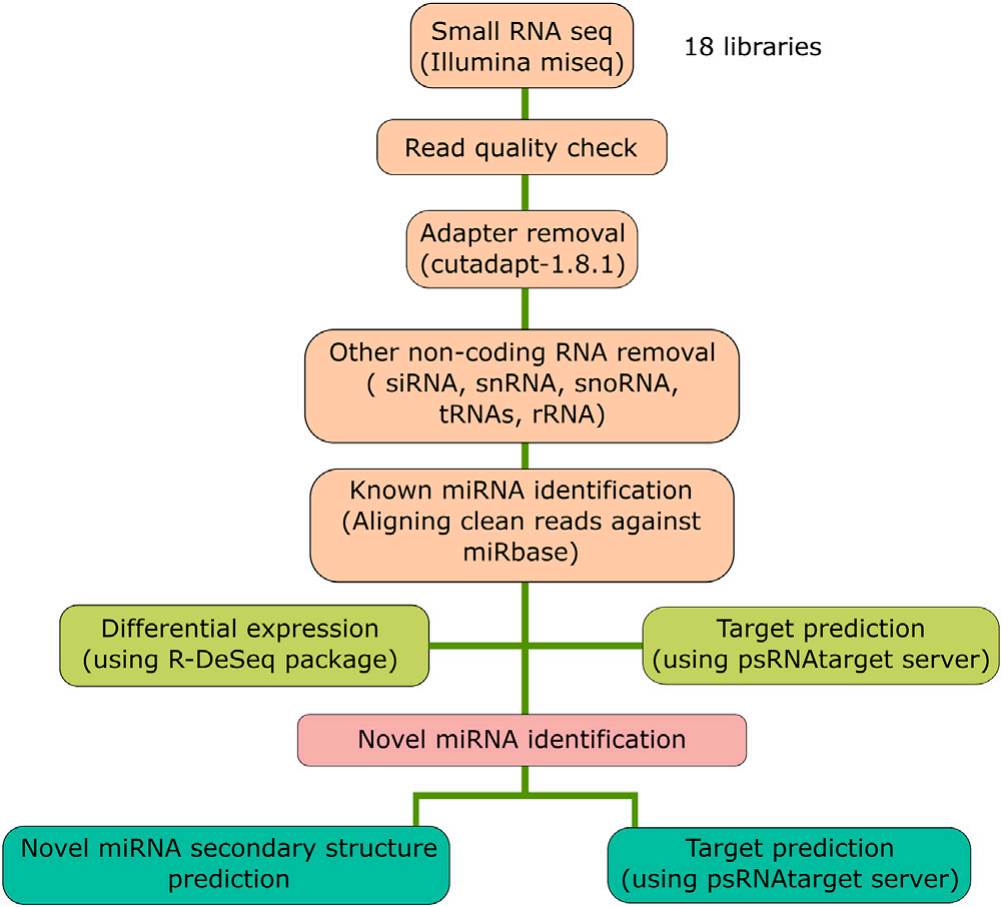
Schematic representation of the bioinformatics analysis pipeline. The flow chart represents the steps followed for the small RNA-sequencing data analysis for the 18 libraries.

The sRNA-sequencing data were analyzed by a set of criteria as described in Figure 1. Initially, the quality of the raw sequencing reads was checked by FastQC (Andrews, 2010) followed by removal of adapter sequences and low-quality bases using Cutadapt (v1.8.1) (Martin, 2011). The quality-filtered clean reads were converted to a non-redundant sequence dataset using the Perl script (Wall, 1994) from the miRGrep tool (Li et al., 2013). These unique read datasets were fed to Bowtie2 aligner (v2.1.0) (Langmead and Salzberg, 2012) for removing other non-coding RNA fragments [transfer RNA (tRNA), ribosomal RNA (rRNA), small nuclear RNA (snRNA), and small nucleolar RNA (snoRNA)]. The retained reads were used to align using Bowtie (v0.12.9) (Langmead et al., 2009) to known rice precursor and mature miRNA sequences downloaded from miRbase (Kozomara et al., 2019) having a maximum of two mismatches. Differential expression (DE) analysis was performed to find out the *R. solani*-responsive miRNAs using the DESeq (v3.0.2) tool (Anders and Huber, 2010) provided in the R program (R Core Team, 2021). The analysis was carried out in binomial mode, and it generated both the heat map and MA-plot. The top ten differentially expressed miRNAs (fold change ≥2 and ≤−2) were extracted by considering the log_2_ fold change value.

### Novel miRNA Prediction

The unaligned reads to either mature or precursor miRNA sequences were extracted for the prediction of putative novel miRNA sequences using the Mireap (v0.2) program (Li et al., 2012). They were further filtered with additional specific criteria for their identification as potential novel miRNAs (Kumar et al., 2017).

1) Length of the miRNA should range between 20 and 26 nt.
2) Minimum length of the pre-miRNA sequence should be 40 nt.
3) The miRNA sequence should be present on one arm of the pre-miRNA in order to form a hairpin secondary structure.
4) The minimum free energy index (MFEI) value of the pre-miRNA secondary structure should be less than -70 so as to differentiate the miRNA from rRNA, tRNA, snRNA, and snoRNA.
5) Mature miRNAs should not have more than four mismatches with the miRNA* sequence.
6) The miRNA sequence arising from the pre-miRNA region should not have any loops.
7) By considering the temporal expression and *R. solani*-induced miRNA biogenesis, the putative miRNA candidate should have at least 2 read count in any dataset.

### cDNA Conversion

DNase-treated RNA was converted into complementary (c)DNA either by poly-A tailing method as described in Balcells et al., 2011 or by miRNA-specific cDNA conversion using miRNA-specific stem-loop (hairpin) primers as per the protocol mentioned in Adhikari et al. (2013) (Supplementary Table S1). Briefly, *E. coli* poly-A polymerase (New England Biolabs, Ipswich, MA, United States) and RevertAid Reverse Transcriptase (RT) (Thermo Scientific, Waltham, MA, United States) were used for RNA poly-A tailing and cDNA conversion. The reaction was set up by mixing 1 μl 10X *E. coli* poly(A) polymerase buffer, 0.2 μl *E. coli* poly(A) polymerase (5,000 U/ml) (New England Biolabs, Ipswich, MA, United States), 1 mM ATP, 10 μM RT primer, 1 mM dNTP mix, and 0.5 μl M-MuLV Reverse transcriptase (200,000 U/ml) (New England Biolabs, United States) along with the RNA template (∼50 pg) in nuclease-free water (Ambion, Thermo Scientific, Waltham, MA, United States). For the cDNA conversion with the stem-loop RT primers, the reaction was performed in a two-way process. In the first step, 2 μl (∼10 pg/μl) of the RNA sample was mixed with miRNA-specific hairpin primer (10 p.m.) and incubated for 5 min at 70°C. Consequently, the incubated sample was mixed with the following components: 5X reaction buffer, 10 μM dNTP, 0.4 μl ribolock RNase inhibitor, 1 μl RevertAid RT (200 U/μl), and nuclease-free water (Ambion, Thermo Scientific, Waltham, MA, United States). This mixture was placed at 42°C for 1 h. As a negative control, another reaction was set up with all the components except RT.

mRNA-specific cDNA conversion was performed with ∼1 μg of DNase-treated total RNA using the RevertAid first-strand cDNA synthesis kit (Thermo Scientific, Waltham, MA, United States) by following the manufacturer’s protocol.

### RT-PCR

RT-PCR was performed for the selected miRNA or mRNA candidates either by poly-A tailing followed by oligo-dT-primed or random-primed cDNA conversion method to confirm the amplification of the target sequence as mentioned in Balcells et al., 2011 and Kumar et al., 2017. For each primer, annealing temperature (T_A_) was optimized to obtain the expected band in PCR for 36 cycles which was confirmed by 1.5% agarose gel electrophoresis.

### Semi-Quantitative PCR

It is followed as per RT-PCR protocol as mentioned previously in Section 2.7 but with 24 cycles.

### Stem-Loop RT-PCR

Stem-loop RT-PCR (SL-RT-PCR) was performed with and without the use of reverse transcriptase-converted miRNA-specific cDNA as described in Adhikari et al., 2013.

### qRT-PCR

qRT-PCR was performed using fluorescent dye SYBR^®^ Green Master Mix (Roche Diagnostics, GmBH, Germany) in a QuantStudio™ 1 real-time PCR system (Thermo Scientific, Waltham, MA, United States) as mentioned previously (Kumar et al., 2017). cDNA (1,100 ng/μl), 2X SYBR^®^ Green Master Mix (10 μl) along with 1 μl each forward and reverse primers (10 pmol/μl each) were mixed in a 20 µl reaction, and each qRT-PCR reaction was carried out in three technical replicates. The fold difference was measured using the 2^**−**ΔΔC**T**^ method (Livak and Schmittgen, 2001), and the standard error was calculated as followed in Mangrauthia et al., 2017.

### MiRNA Target Prediction, GO Analysis, and Their Quantification

The psRNATarget web tool (Dai et al., 2018) with default parameters was used to predict the miRNA target sequences by feeding the *O*. *sativa* transcriptome data from Ensembl Plants (https://plants.ensembl.org/index.html). Functional enrichment analysis like GO analysis (Ashburner et al., 2000) was performed to classify the miRNA target genes into molecular function, cellular component, and biological processes. The primers were designed (Supplementary Table S1) specific to miRNA target genes which were validated by RT-PCR, semi-quantitative PCR, and qRT-PCR as per the aforementioned protocols.

### 5′ RLM-RACE-PCR

5′RLM-RACE-PCR was carried out to find out the miRNA binding and cleavage site in the target mRNA. FirstChoice^®^ RLM-RACE Kit (Thermo Scientific, Waltham, MA, United States) was used for performing 5′RLM-RACE as per the manufacturer’s protocol with some modifications by excluding the calf intestine alkaline phosphatase (CIP) and tobacco acid pyrophosphatase (TAP) treatment. A volume of 1 µl 5′ RACE Adapter RNA (0.3 μg/μl) was ligated to 5′-monophosphate-containing RNA using 2 µl T4 RNA Ligase (2.5 U/µl) in a 10 µl reaction mixture at 37°C for 1 h. A volume of 2 µl ligated RNA mix was used for reverse transcription using 1 µl of gene-specific reverse primer (10 µM), 4 µl dNTPs (2.5 mM each), 1 µl RNase inhibitor (10 U/µl), and 1 µl RevertAid RT in a 20 µl reaction mixture at 42°C for 1 h. The outer reverse primer (ORP) and inner reverse primer (IRP) were designed specifically to cleave the mRNA sequences (Supplementary Table S1), and outer 5′ RLM-RACE PCR followed by inner 5′ RLM-RACE PCR was performed as mentioned in the kit.

### Cloning

The expected 5′RLM-RACE product was gel eluted and cloned into pTZ57 R/T (InsTAclone PCR Cloning kit, Thermo Scientific, Waltham, MA, United States) vector as per manufacturer’s instructions, confirmed by restriction digestion using *EcoR*I and *Hind*III (Thermo Scientific, Waltham, MA, United States) and Sanger sequencing. Similarly, to confirm the NmiR1 origin, specific primers (Supplementary Table S1) were used to amplify the precursor miRNA region by the rice genomic DNA as a template. The expected product was cloned in the pGEMT-Easy vector and confirmed by *Eco*RI, sequence-specific *Not*I, *Ssp*I, and by sequencing.

## Results

### Small RNA Sequencing and Data Analysis

**FIGURE 2:**
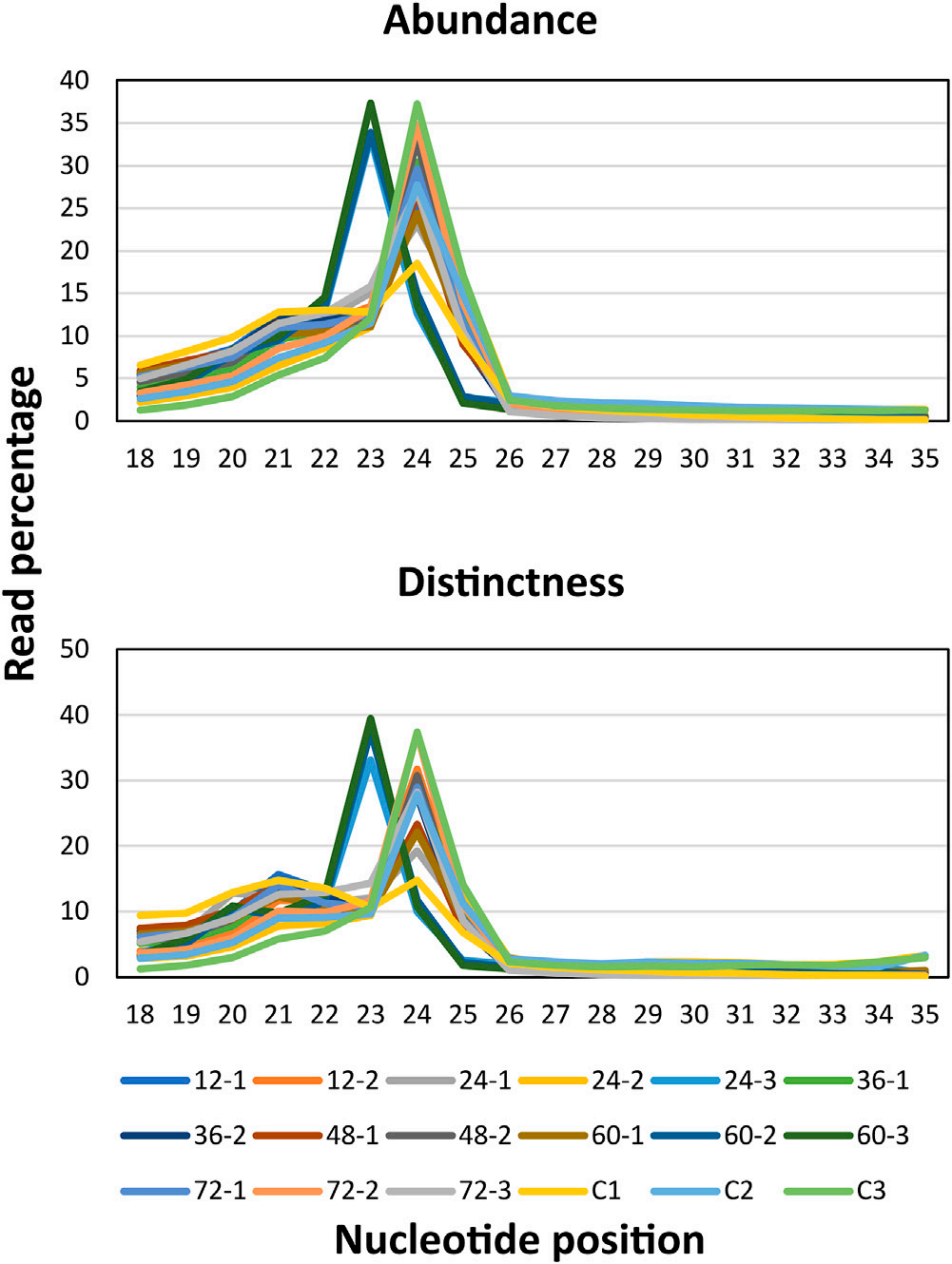
Read abundance. The sRNA-sequencing reads were subjected for removal of the low-quality reads and adapter sequences. The clean reads were plotted by taking read length on X-axis and the percentage of the clean reads on Y-axis to observe the read abundance which highlighted all the reads falling within the range of 20–26 nt. Read distinctness. The length of the total clean unique reads was plotted by taking the read length on X-axis and the percentage of the clean unique reads on Y-axis.

MiRNAs regulated the function of target genes either by cleaving or by blocking the protein translation via binding to the 3’ untranslated region (UTR) of the target mRNAs. In addition, miRNAs that assist or curtail the survival of plant pathogens in the host as well as the mechanisms by which plant pathogens modulate the levels of miRNAs in order to multiply in the host are to be deciphered yet (Weiberg et al., 2013; Lin et al., 2016; Chopperla et al., 2020; Mathur et al., 2020; Molla et al., 2020; Wang and Dean, 2020; Wenlei et al., 2020). Therefore, in order to identify the rice miRNA profiles upon *R. solani* infection, large-scale time series (0, 12, 24, 36, 48, 64, and 72 hpi) sequencing was conducted. A total of ∼435 million paired-end clean reads were obtained from all the sequencing libraries combined after removing the adapters and other low-quality sequences. The quality (Phred score ≥ Q30) of the sequencing libraries ranged from 93.35 to 98.80%, with an average of 95.77%, which indicates reliable data quality (Supplementary Table S2). The cleaned quality reads were further processed to filter out the rRNA, snRNA, snoRNA, tRNA, small interfering RNAs (siRNAs), and other RNA fragments that resulted in retaining ∼38 million clean unique reads of 17–35 nt length (Table 1, Supplementary Table S2). The graph showed the percentage of clean unique reads (17–35 nt length) from each sequencing dataset and showed read abundance and distinctness spiked at 23 nt and 24 nt, respectively (Figure 2 **).**

**TABLE 1:** sRNA-sequencing data statistics. The total number of raw reads and clean unique reads after quality filtration are listed for each sequencing dataset. The total number of known miRNAs and Mireap-predicted novel miRNAs for each dataset are shown in the table.

Sample ID	Total read	Clean unique read (17–35 nt length)	Number of known mature miRNAs	Mireap-predicted number of novel miRNAs
C1	25,877,946	1,059,960	278	207
C2	30,559,927	3,496,657	331	46
C3	26,035,547	673,760	211	38
12-1	28,805,547	1,910,760	322	287
12-2	35,868,528	3,569,456	322	613
24-1	11,632,558	1,359,004	291	68
24-2	16,826,644	1,682,554	303	119
24-3	32,176,609	2,205,186	331	471
36-1	19,700,015	1,563,439	312	281
36-2	16,559,235	2,435,640	293	146
48-1	12,600,997	909,225	269	40
48-2	15,259,729	1,786,581	311	80
60-1	14,392,841	486,151	253	24
60-2	59,806,227	3,981,426	392	402
60-3	16,800,136	2,368,239	361	320
72-1	22,094,208	2,332,185	316	120
72-2	21,876,105	3,825,080	331	257
72-3	28,568,850	3,313,148	346	183
Total	435,441,649	38,958,451	—	

### *R. solani*-Induced miRNA Expression Dynamics in Rice

**FIGURE 3:**
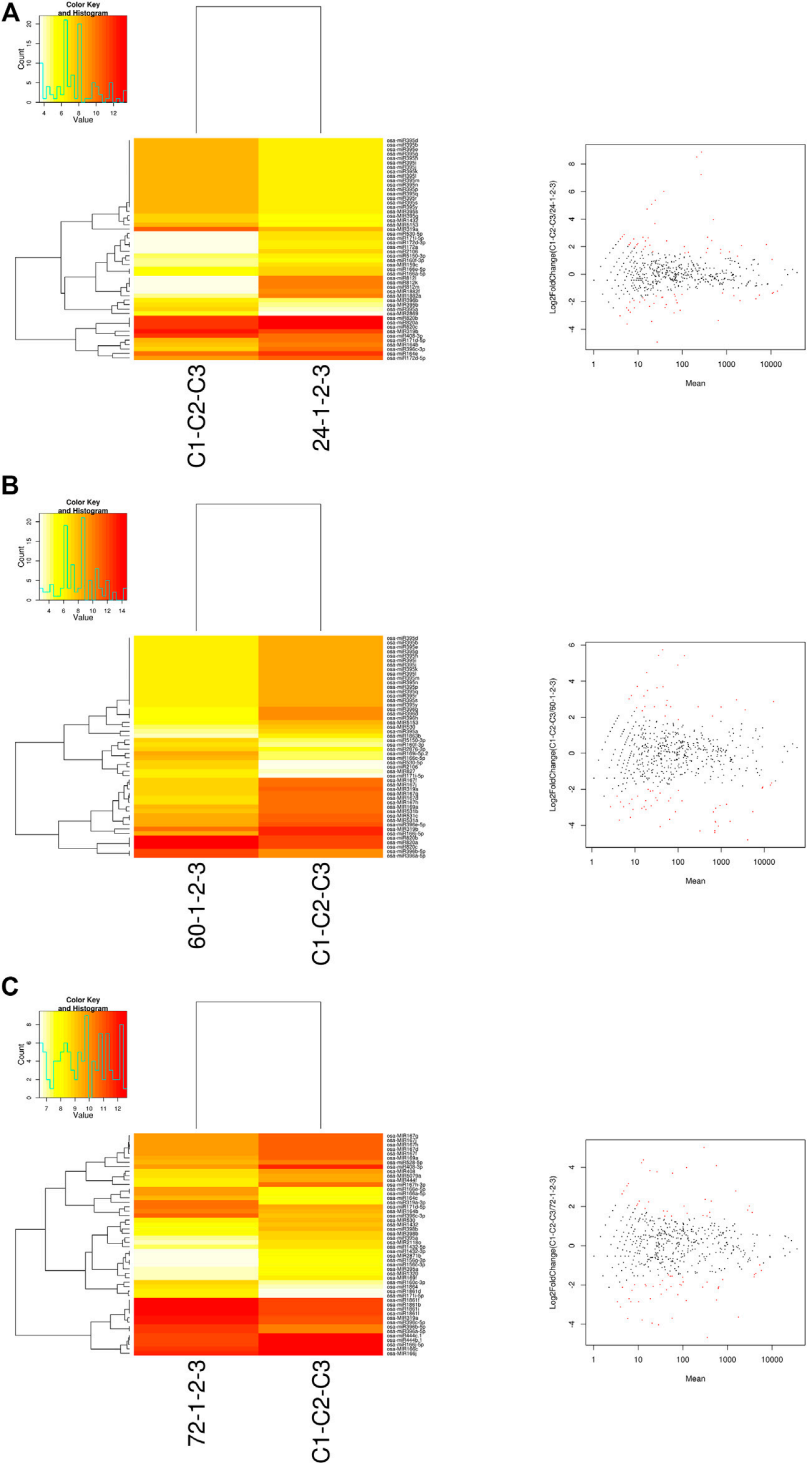
Heat map and MA plots. The heat maps were generated for the control sample versus the sample at each of the infected time points such as **(A)** 24 hpi, **(B)** 60 hpi, and **(C)** 72 hpi with the normalized read count data matrix using a g-plot heat map. The plot shows the hierarchical clustering of both samples (yellow shows low-level expression; red shows high-level expression). The log_2_ fold change expression values were calculated by DESeq in binomial mode. The MA plots were generated to identify the ≥2-fold (highly expressed) and ≤-2-fold change (low expressed) miRNAs. X-axis represents the difference between the log_2_ fold change values, and Y-axis represents the average of the log_2_ fold change values. The red dots in MA-plots represent the potential miRNAs expressed upon *R. solani* infection.

**FIGURE 4:**
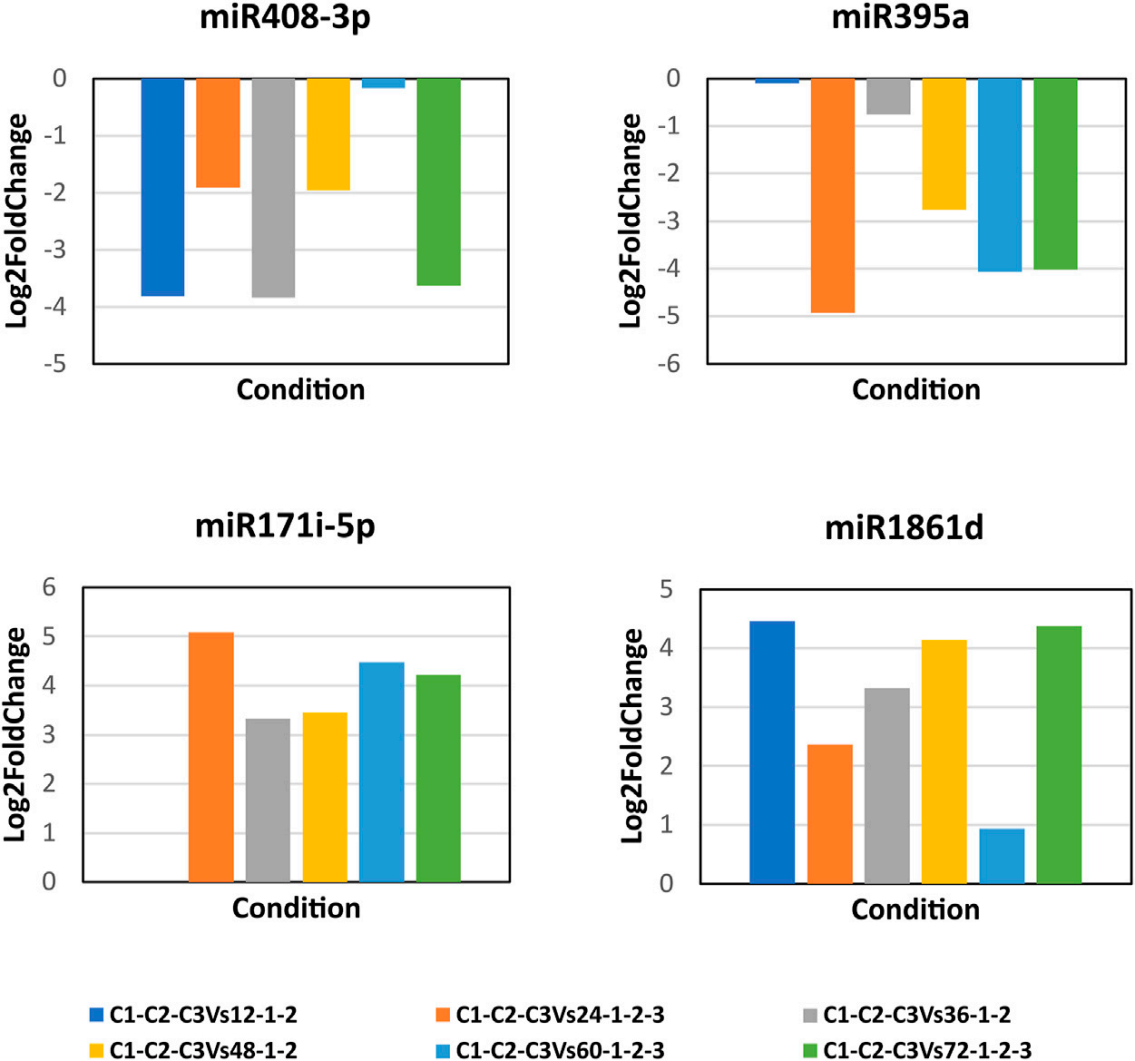
Bar plots of the candidate miRNA log_2_ fold change expression values. The bar plots represent the log_2_ fold change expression values of the candidate miRNAs at 12, 24, 36, 48, 60, and 72 h post-infection of *R. solani* with respect to control samples. X-axis represents the condition (control vs. infection time point), and Y-axis represents the log_2_ fold change expression values calculated by DESeq. miR408-3p and miR395a show the downregulation, whereas miR171i-5p and miR 1861d show the upregulation upon *R. solani* post-infection.

The DE analysis between the controls with each of the infected samples separately generated the log_2_ fold change values for each combination. Heat maps and MA plots of the DE analysis were created for each of the six combinations of control versus infected libraries (Figure 3; Supplementary Figure S1). By considering the log_2_ fold change values, we extracted the top up- and downregulated miRNAs from all six combinations of DE results (Supplementary Table S3). The bar plots of the log_2_ fold change values of the five selected known candidate miRNAs at different time points of *R. solani*-infected plant were generated with respect to control plants. miR171i-5p and miR 1861d had shown upregulated expression, whereas miR395a and miR408-3p exhibited downregulated expression upon *R. solani* infection (Figure 4).

### sRNA-Sequencing Data Validation

It is observed that selected miRNAs such as miR395a, miR408-3p, miR171i-5p, and miR 1861d were differentially modulated ranging between minimum 1-fold and maximum 5-fold. RT-PCR was carried out using miRNA-specific primers with cDNA from the control and infected plants at various time points of post-infection. The experimental data yielded accurate miRNA-specific amplification with all the tested primers (Supplementary Figure S2). Semi-quantitative PCR was carried out for miR395a and miR408-3p for control, 48 hpi, and 72 hpi samples which showed differential expression (Supplementary Figure S3).

**FIGURE 5:**
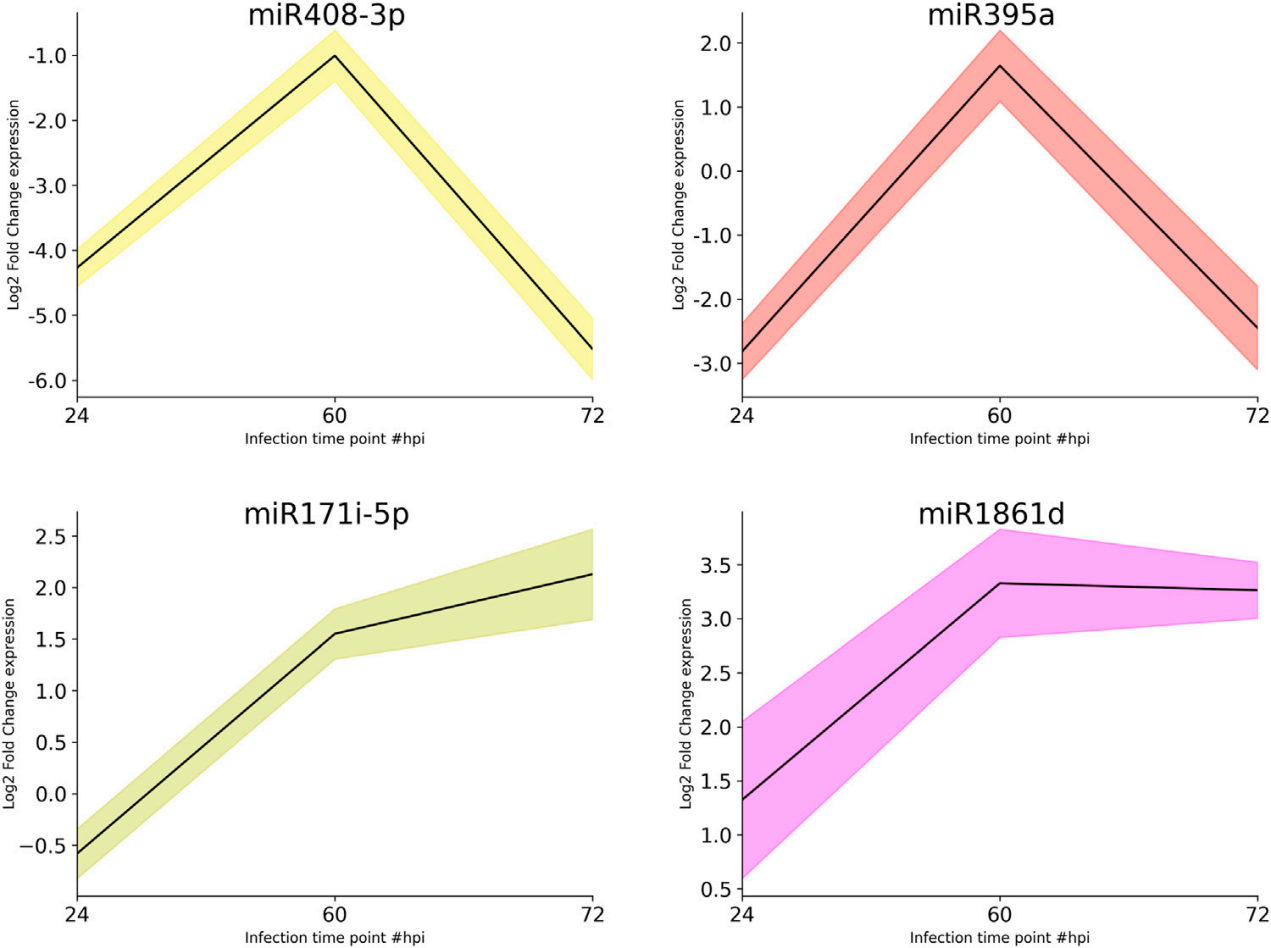
qRT-PCR. The total RNA was isolated from the control and *R. solani*-infected plants at various time points. Initially, RT-PCR was performed to assure the miRNA primer quality and specific amplification. After ensuring the primer quality, qRT-PCR was performed to quantify miR395a, miR408-3p, miR171i-5p, and miR 1861d. The bar plots represent the approximate differential expression levels of the candidate miRNAs generated by the qRT-PCR technique. The differential expression levels were determined in fold difference using the 2^−∆∆CT^ method for the *R. solani*-infected rice plant at various time points, namely, 24, 60, and 72 hpi, with respect to the uninfected plant grown under controlled conditions.

Subsequently, qRT-PCR was carried out to determine the expression dynamics of candidate miRNAs in control and infected plants at different time points (24, 60, and 72 hpi) post infection. miR395a and miR408-3p showed a ∼3- and >4-fold downregulation, respectively, at 24 hpi and ∼2.5- and ∼6-fold downregulation, respectively, at 72 hpi although miR395a displayed a slight upregulation (1.5-fold) at 60 hpi with respect to the control plant. Similarly, miR171i-5p and miR 1861d showed a maximum of 2- and 3-fold upregulation, respectively, at 72 hpi even though miR171i-5p exhibited a slight downregulation (∼0.5-fold) at 24 hpi (Figure 5). This further suggests that the qRT-PCR expression plots of miR395a, miR408-3p, miR171i-5p, and miR 1861d are in line with the NGS-analyzed data.

### Putative Novel Rice miRNA Identification and Validation

**FIGURE 6:**
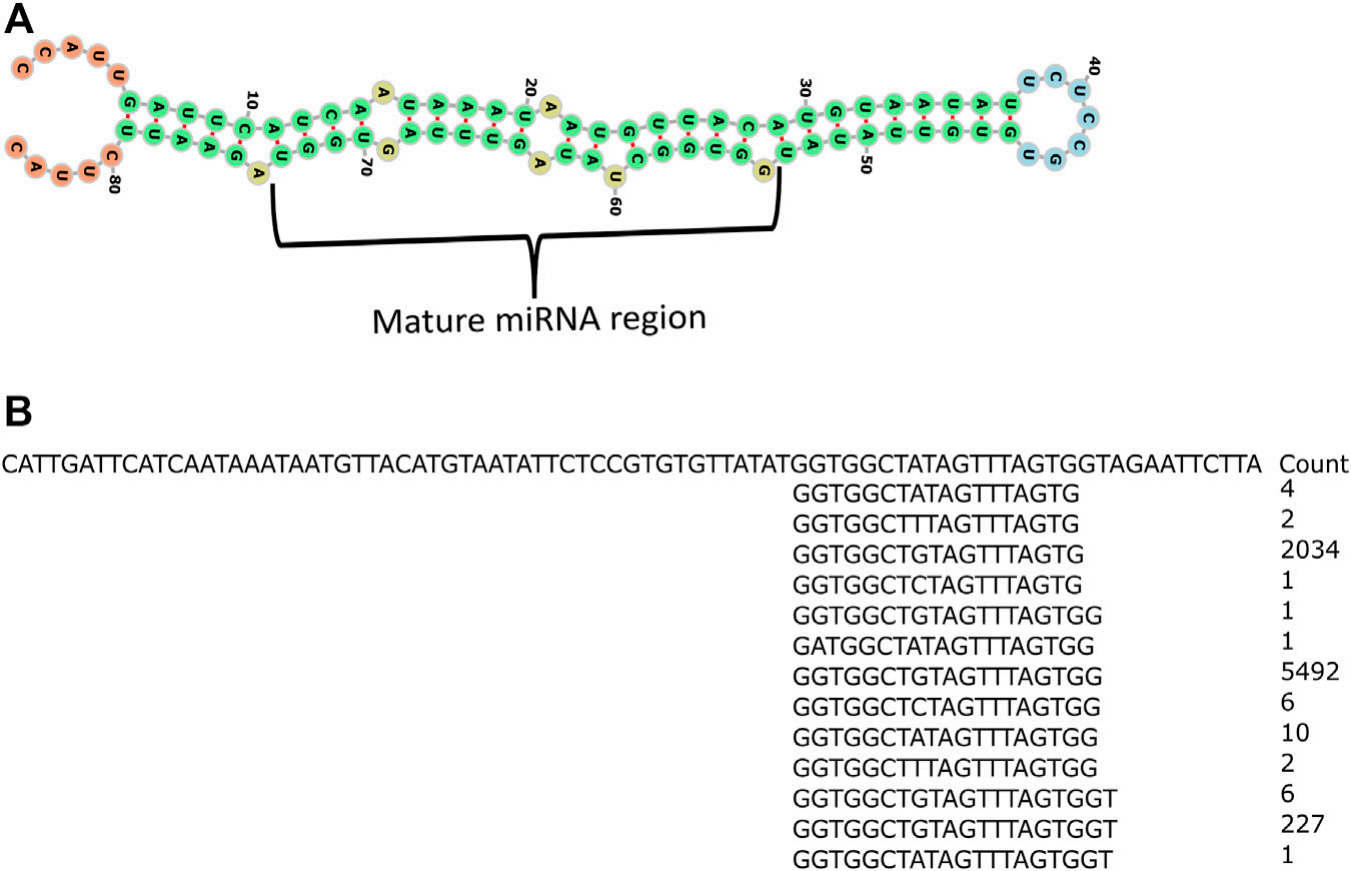
Secondary structure of the NmiR1 precursor sequence and read alignment. **(A)** RNA fold predicted possible minimum energy secondary structure of the NmiR1 precursor sequence with perfect alignment of the mature miRNA region to its star miRNA. **(B)** Read alignment of 24 hpi third biological replicate sequencing data with read count.

The putative novel miRNAs were identified by extracting the sequencing data which were neither aligned to known mature miRNA nor known precursor miRNA sequences. This prompted us to verify further the genuineness and reliability of such candidates. The unaligned sequencing data were used as an input for Mireap (v0.2) (Li et al., 2012), a computational tool to predict the putative novel miRNAs. Mireap has predicted between 207, 46, 38, 287, 613, 68, 119, 471, 281, 146, 40, 80, 24, 402, 320, 120, 257, and 183 novel putative miRNAs for all the 18 libraries (Table 1). We selected one putative novel miRNA (NmiR1) out of all Mireap-predicted miRNAs based on its read count and found consistent expression in all the biological replicates. The precursor sequence of the NmiR1 is 84 nt length and its secondary structure with 0.70 MFEI value. Figure 6A shows the mature miRNA region having the perfect base pairing with its *miRNA region present in another arm. The NmiR1-aligned sequencing data at 24 hpi (third biological replicate) show a total of 13 isomiRNAs (isomiRs) with few base changes having a read count distributed between 1 and 5492 (Figure 6B). The GC contents are 45 and 29% for both NmiR1 and its precursor sequence, respectively. It is also found that the NmiR1 precursor sequence is present in two different regions on chromosome 11 of rice at 19781155–19781238 nt and 19774521–19774604 nt genome locations (Supplementary Table S4).

**FIGURE 7:**
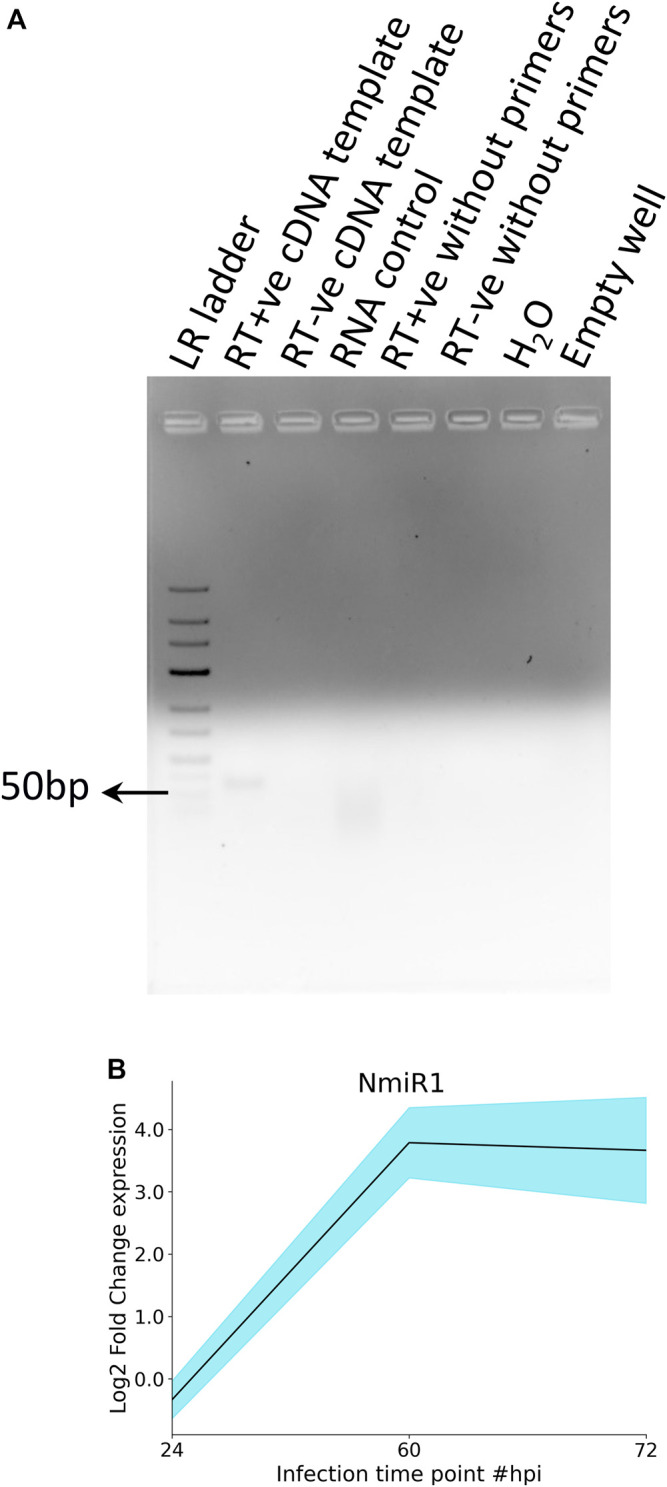
RT-PCR and qRT-PCR analysis of NmiR1. **(A)** In order to confirm the novel miRNA prediction, specific primers were designed for putative novel miRNA (NmiR1) and subjected to RT-PCR to visualize the expected amplicon (50 bp), whereas negative controls (RNA, H_2_O) did not yield any product. **(B)** qRT-PCR differential expression analysis shows the upregulation of NmiR1 at 60 hpi and 72 hpi of *R. solani* inoculation with respect to control rice.

NmiR1 validation was performed by SL-RT-PCR by miRNA-specific hairpin primer-derived cDNA that yielded the expected size amplicon (Figure 7A, lane 2), whereas none of the different negative controls and water control yielded any product (Figure 6A). Furthermore, qRT-PCR was performed for studying the NmiR1 expression dynamics between control and *R. solani*-infected plants. NmiR1 expression was found to be downregulated at 24 hpi (early infection), but a steep upregulation of above 10-fold was observed at 60 hpi and 72 hpi (Figure 7B). Using specific primers, precursor-NmiR1 (pre-NmiR1) was cloned and sequenced which will be used to further decipher the functional significance (Supplementary Figure S6).

### MiRNAs Target Determination, GO Analysis, and Quantification

**FIGURE 8:**
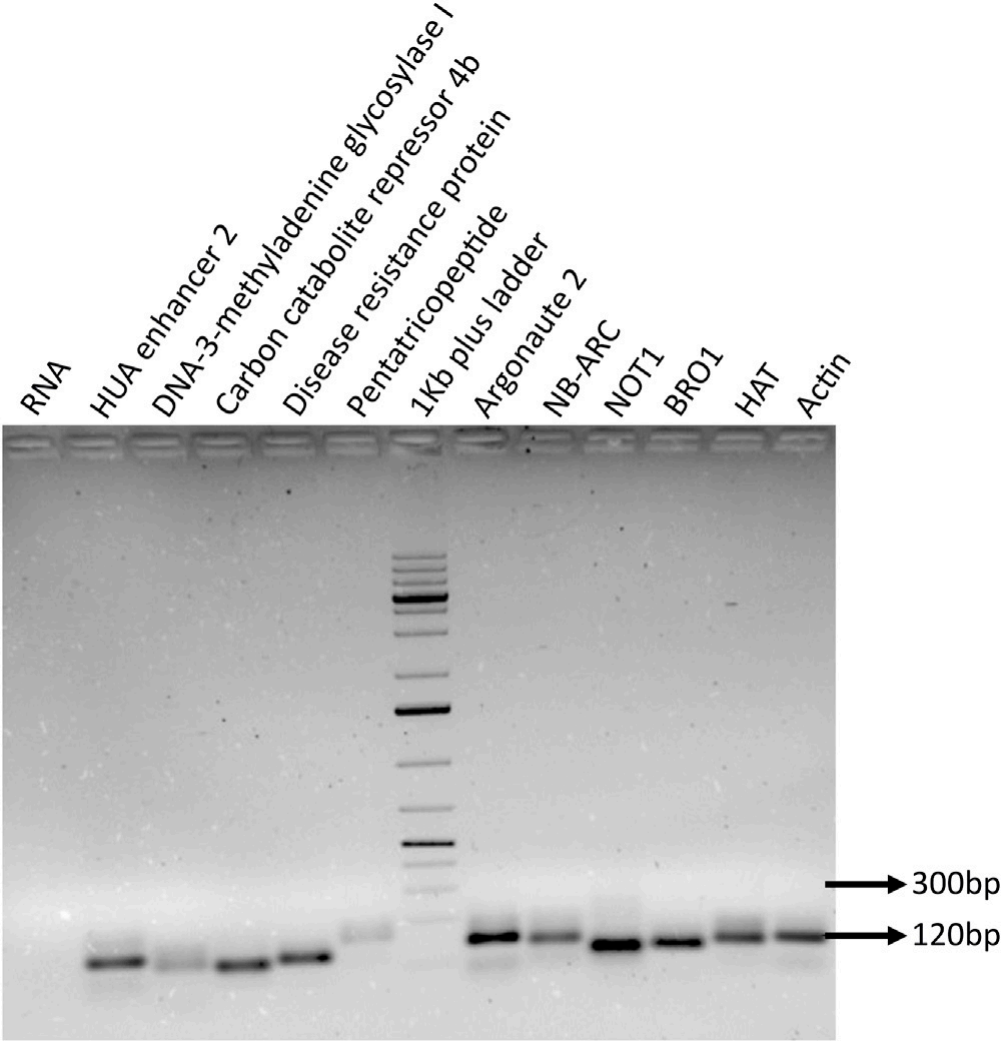
PCR amplification of selected target genes. The miRNA target genes were identified by performing *in silico* analysis, and specific target was selected based on the miRNA-binding ability and score. The specific primers were designed for the selected genes, and primer quality and precise amplicon were ensured by performing RT-PCR along with negative RNA and internal control actin.

Both small RNA-sequencing data and qRT-PCR results confirm the differential modulation of miR395a, miR408-3p, miR171i-5p, and miR 1861d in *R. solani*-infected plant. Furthermore, an attempt was made to predict the miRNA-targeted genes by both psRNAtarget web server and GO analysis in which a total of 10 target genes (Table 2) were identified for miR395a, miR408-3p, miR171i-5p, and miR 1861d altogether. Based on their possible role in plant immune mechanisms, primers were designed (Supplementary Table S1), and RT-PCR was carried out using these gene-specific primers which yielded the expected amplicon corresponding to each target gene **(** Figure 8). Similarly, semi-quantitative PCR was carried out with miRNA-specific target genes (DNA-3-methyladenine glycosylase I, carbon catabolite repressor 4b (CCR4b), HUA enhancer 2, Argonaute2, and pentatricopeptide) which showed differential expression at different time points post *R. solani* infection (Supplementary Figure S4).

**TABLE 2:** List of selected target protein-coding genes for the miR395a, miR408-3p, miR171i-5p, and miR 1861d. The target prediction analysis was performed by the psRNAtarget web server. A total of 10 targets were selected; among them 4 for miR395a, 3 for miR408-3p, 2 for miR171i-5p, and 1 for miR1861d were chosen.

Gene name	Transcript	Description
miR395a targets		
Os08t0170700	Os08t0170700-00	Disease resistance protein domain-containing protein
Os12t0109300	Os12t0109300-01	Pentatricopeptide repeat domain-containing protein
OsAGO2	Os04t0615700-01	Argonaute and Dicer protein and PAZ domain-containing protein
Os12t0565100	Os12t0565100-01	NB-ARC domain-containing protein
miR408-3p targets		
Sdt97	Os06t0649800-01	Similar to DNA-3-methyladenine glycosylase I
OsDSHCT	Os11t0176200-01	Similar to HUA enhancer 2
OsCCR4b	Os03t0166800-01	Carbon catabolite repressor 4b, exonuclease-endonuclease-phosphatase (EEP), and mRNA deadenylation
miR171i-5p targets		
OsNOT1	Os10t0556700-01	Not1 protein (CCR4-Not1 complex component and mRNA deadenylation)
Os04g0527400	Os04t0527400-02	BRO1 domain-containing protein
miR 1861d targets		
Os05t0102300	Os05t0102300-01	HAT dimerization domain-containing protein

**FIGURE 9:**
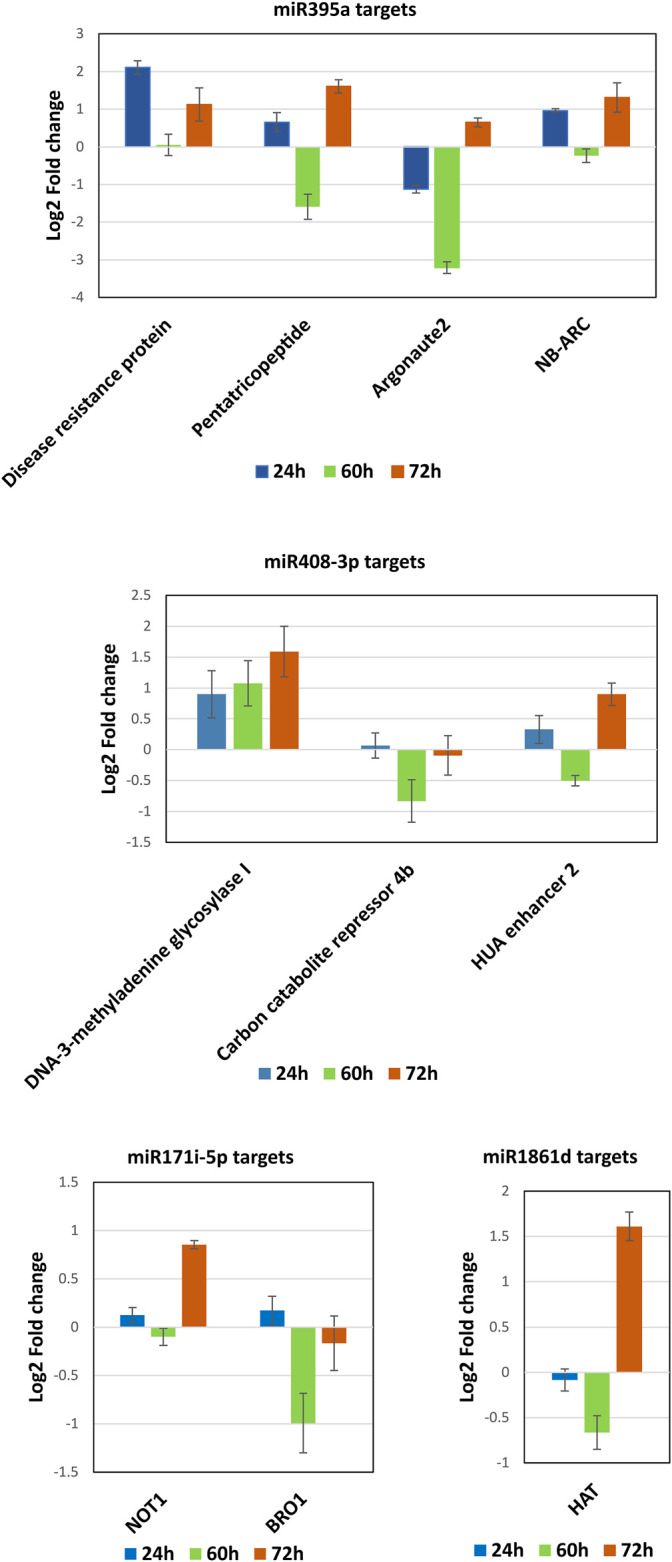
MiRNAs target quantification. The possible mRNA targets for the miRNAs such as miR395a, miR408-3p, miR171i-5p, and miR 1861d were subjected to qRT-PCR to quantify and correlate the expression between miRNA and mRNA at 24, 60, and 72 hpi of *R. solani* infection with respect to control samples.

qRT-PCR was performed to quantify the selected miRNA target genes between control and infected plants. The DE analysis of miR395a targets such as disease resistance protein, pentatricopeptide, and NB-ARC showed their upregulated expression at 24 hpi except for Argonaute2 which displayed downregulated expression as compared to control rice (Figure 9 **).** However, all the targets for miR395a were upregulated at 72 hpi (>0.01-<2-fold) and downregulated at 60 hpi (>0.2-∼3.2-fold) (Figure 9 **).** For miR408-3p target genes, DNA-3-methyladenine glycosylase I showed a gradual increase in expression from 24 hpi to 72 hpi (<1-∼1.5-fold), whereas CCR4b and HUA enhancer 2 exhibited ∼0.8-fold and 0.5-fold downregulated expression, respectively, at 60 hpi. Expression of CCR4b was, though, comparable with control plants at both 24 hpi and 72 hpi. However, the other miR408-3p target, HUA enhancer 2, displayed upregulated expression at both 24 hpi and 72 hpi (Figure 9 **)**. The target mRNAs of miR171i-5p such as NOT1 and BRO1 had expressed almost equal but opposite expressions at 24, 60, 24, and 72 hpi, respectively. Nonetheless, both the targets had also exhibited reciprocal expression but at two different time points; ∼1-fold upregulation of NOT1 at 72 hpi, whereas 1-fold downregulation of BRO1 at 60 hpi (Figure 9). The candidate mRNA, HAT, targeted by miR 1861d showed an increase in downregulated expression from 24 to 60 hpi (up to 0.7-fold) but displayed upregulation at 72 hpi as compared to mock-infected rice plants (Figure 9). Pentatricopeptide, DNA-3-methyladenine glycosylase I, and BRO1 expressions show an inverse correlation to their respective miRNA’s expression such as miR395a, miR408-3p, and miR171i-5p, respectively, in all the three time points.

### Binding Target mRNA and Its Cleavage Site Determination

**FIGURE 10:**
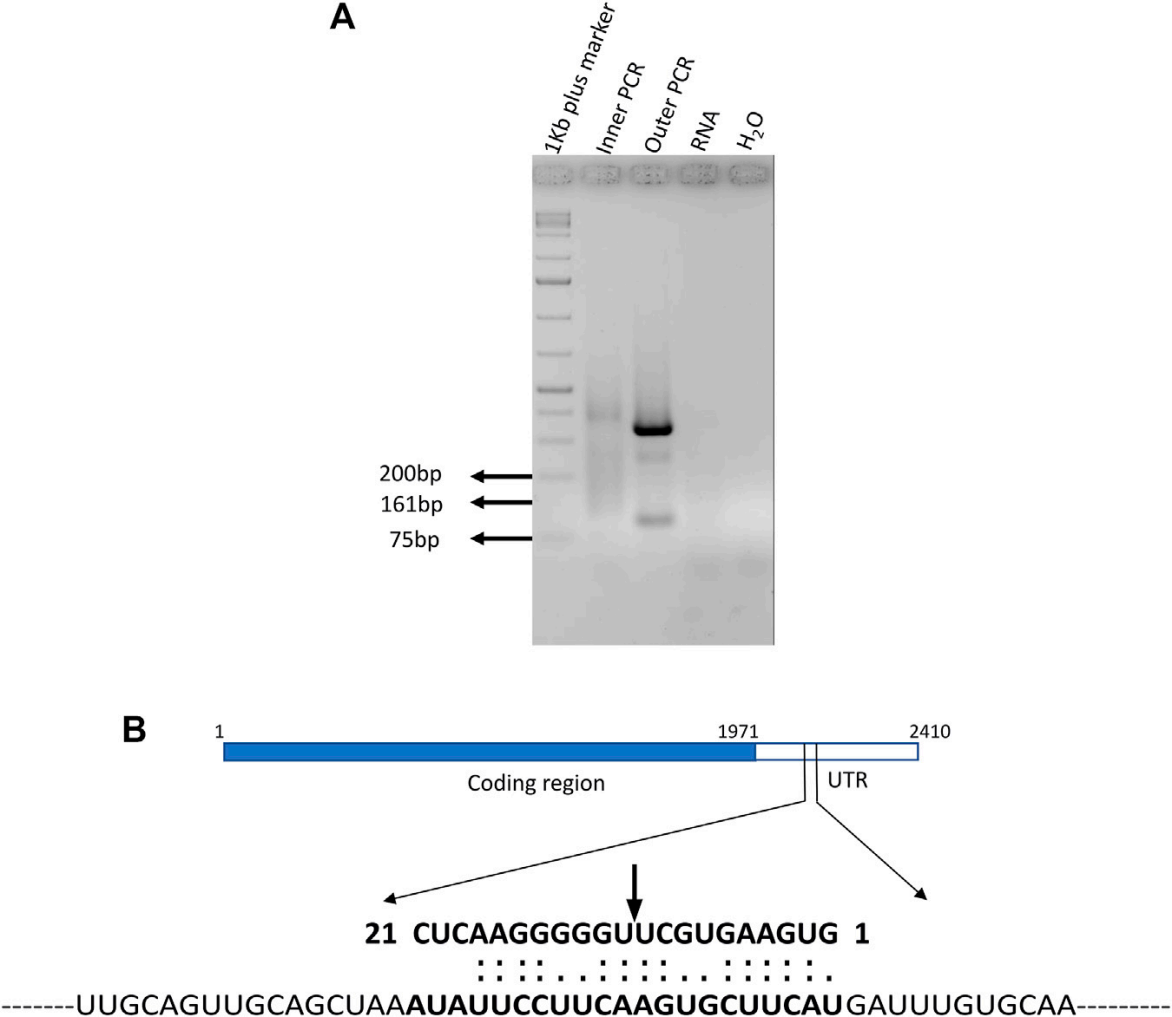
MiR395a putative target gene pentatricopeptide 5′ RLM-RACE and psRNAtarget-predicted binding region. **(A)** 5′ RLM-RACE was performed with adapter and gene-specific primers and the expected product of 161 bp was observed in inner PCR. **(B)** psRNAtarget predicted the miR395a-binding region on the pentatricopeptide mRNA sequence which shows miR395a binds at the 2155–2175 nt region of the PPR mRNA, and the possible cleavage site is present between 10th and 11th bases.

The RT-PCR and qRT-PCR results demonstrate that miR395a, miR408-3p, and miR171i-5p target gene (pentatricopeptide, DNA-3-methyladenine glycosylase I, and BRO1) expressions are inversely correlated with the miRNA expression which hints that it may be regulated by the miRNA. Therefore, specific primers were designed to determine the miRNA binding and cleavage site by 5′RLM-RACE for these aforementioned target genes. The outer and inner primers were designed, and 5′ RLM-RACE was performed for all the selected target mRNAs (Supplementary Table S1). The cDNA was produced using the target mRNA-specific reverse primer after ligating with RNA adapter at the 5′ end of DNase-treated RNA without subjecting to CIP- and TAP-treatment. The restriction analysis had shown the presence of *Mse*I site in the expected inner RACE product of the pentatricopeptide mRNA which was subsequently confirmed by *Mse*I digestion. The digested product was observed to have the expected band size of 77 and 84 bp, including the RACE adapter sequence (Supplementary Figure S5). Further confirmation was performed by cloning many expected sized products for the selected miRNAs followed by Sanger sequencing to reveal the sequence. Out of three clones, only the miR395a target was shown with an expected product size of 161 bp (Figure 10A **).** There was no amplification found when RNA was used as a template, which further confirmed the authenticity of the outer and inner PCR (Figure 10A **)**. MiR395a was predicted to bind at the 2155–2175 nt region of the pentatricopeptide mRNA, and the possible cleavage site was shown between the 10th and 11th base of the binding region (Figure 10B). The selected pentatricopeptide mRNA was predicted to localize the chloroplast according to ChloroP (http://www.cbs.dtu.dk/services/ChloroP/) (Emanuelsson et al., 1999) and TargetP (http://www.cbs.dtu.dk/services/TargetP/) (Armenteros et al., 2019) prediction tools.

## Discussion

*R. solani* causing ShB in rice is a highly destructive disease leading to high yield losses worldwide. Several efforts were made to develop the ShB-resistant rice lines using various genes or quantitative trait loci (QTLs) by either breeding or genetic transformation, but none of them were able to produce a good ShB-resistant line (Molla et al., 2020). This could be due to the quantitative trait of the ShB resistance mechanism (Molla et al., 2020). Little efforts were made to identify genes/QTLs which produce ncRNAs such as long ncRNAs (lncRNAs), siRNAs, circular RNAs (circRNAs), and miRNAs. MiRNAs are the key regulator in plant development and disease resistance with very few studies reporting their role during *R. solani* infection in rice (Molla et al., 2020). However, the effectiveness of host-delivered RNA interference (HD-RNAi) technology was comprehended and shown for the first time in ShB-resistant transgenic rice lines that produced siRNA to target *R. solani* homologs of pathogenicity map kinase 1 (*PMK1*) (Tiwari et al., 2017). Recently, Chopperla et al. (2020) conducted the *R. solani*-infected time-series study at 72 hpi for studying the miRNA expression on six rice genotypes which included susceptible and resistant *indica* and aus rice cultivars along with some of the wild rice varieties. It revealed the differential expression of various miRNAs such as Osa-miR1320-5p, Osa-miR156d, Osa-miR159b, Osa-miR166h-3p, Osa-miR166j-5p, Osa-miR169a, Osa-miR396f-5p, Osa-miR397b, Osa-miR398b, Osa-miR528-5p, Osa-miR530-5p, Osa-miR820c, Osa-miR1862d, Osa-miR1876, and Osa-miR2878-5p in rice upon *R. solani* infection. Similarly, Wenlei et al. (2020) had shown a total of 400–450 known and 450–620 novel miRNA expressions across 5, 10, and 20 hpi libraries and showed modulated expression of various miRNAs such as Osa-miR398a, Osa-miR1881, Osa-miR530, Osa-miR444, and Osa-miR812.

In analogy with the previous studies, we have performed the time series small RNA-sequencing profile of *R. solani*-infected rice plants to reveal the miRNA expression dynamics in which our data coincide with Wenlei et al. (2020) in identifying more miRNAs than Chopperla et al. (2020). We have generated the in-depth sequencing data of ∼435 million reads compared to the ∼190 million (Chopperla et al., 2020) and ∼351 million reads (Wenlei et al., 2020). In the current study, we found several miRNAs which were showing varied expression levels but the best four known candidate miRNAs like miR395a, miR408-3p, miR171i-5p, and miR 1861d and one novel miRNA, NmiR1, had shown consistent expression dynamics.

Previously, several reports have shown the novel miRNA prediction and validation in rice under various biotic and abiotic conditions. Sunkar et al. (2008), Jeong et al. (2011), Baldrich et al. (2015), Mutum et al. (2016) Wen et al. (2016), Chopperla et al. (2020), and Wenlei et al. (2020) are a few studies that showed novel miRNAs in rice by high-throughput sequencing data analysis. Similarly, in our study, we have predicted several putative miRNAs from the small RNA-sequencing data upon *R. solani* infection, selected one novel miRNA (NmiR1), and confirmed by SL-RT-PCR. However, further work is required to elucidate the NmiR1 role by gain and/or loss functional studies. Target analysis shows that the NmiR1 can target the defense-related genes such as *WRKY* factors, wound-induced protein (*WIP3*), RAN GTPase-activating protein 1, powdery mildew resistance protein *PM3A*, PPR proteins, methyltransferase, F-box domain-containing protein, and hypersensitive-induced response protein. Apart from these, it was also shown to target plant developmental-related proteins. However, the putative targets should be confirmed by RLM-RACE.

The functional relevance of studied miRNAs like miR395a, miR408-3p, miR171i-5p, and miR 1861d and their targets were emphasized by performing RACE-PCR in order to confirm the miRNA-binding site. It was revealed that miR395a could bind and cleave the pentatricopeptide repeat (PPR) protein-coding mRNA. It was reported that pentatricopeptide (Os12t0109300-01; LOC_Os12g01850) is a high-confidence target for miR395a in rice (Peng et al., 2020). PPR proteins constitute a large gene family in plants which are characterized by tandem repeats of degenerated 35-amino acid (PPR motif) sequence (Small and Peeters, 2000) that forms a pair of anti-parallel alpha-helix structure which allows the binding to RNA strands and majorly involves in post-transcriptional modifications of organellar RNA (Fujii and Small, 2011; Barkan et al., 2012; Yin et al., 2013; Barkan and Small, 2014; Wang et al., 2021). Recently, several studies have also proved that the majority of the PPRs are involved in chloroplast and mitochondrial development and RNA metabolism. For instance, the knockdown of ALS3 PPR protein leads to the disruption of rice chloroplast (Lin et al., 2015). Similarly, several other PPR proteins such as seedling-lethal chlorosis 1 (Lv et al., 2020), pale-green leaf 12 (PGL12) (Chen et al., 2019), white stripe leaf 4 (WSL4) (Wang et al., 2017), thermo-sensitive chlorophyll-deficient mutant 10 (TCD10) (Wu et al., 2016), lovastatin insensitive 1 (LOI1) (Tang et al., 2010), seedling-lethal albino 4 (OsSLA4) (Wang et al., 2018), OsPPR16 (Huang et al., 2020), and OsPPR4 (Asano et al., 2013) were characterized to be involved in chloroplast development and function. Another report has shown that the DUA1 PPR protein is involved in regulating the chloroplast gene expression by interacting with the sigma factor, OsSIG1, in rice (Du et al., 2021). It is well established that PPRs are involved in chloroplast-encoded RNA editing and processing. For the first time, maize PPR, CRP1, was shown to be involved in mono-cistronic *petD* mRNA processing from a polycistronic precursor and cytochrome *f* translation from the chloroplast *petA* transcripts (Barkan et al., 1994; Fisk et al., 1999; Meierhoff et al., 2003). In rice, OsPPR6 was shown to be involved in chloroplast RNA editing and splicing (Tang et al., 2017). The PPR editing is performed by the E and DYW domains at the C-terminus which consists of cytidine deaminase activity (Okuda et al., 2007, 2009; O’Toole et al., 2008; Boussardon et al., 2012; Chateigner-Boutin et al., 2013; Hayes et al., 2013; Schallenberg-Rüdinger et al., 2013; Wagoner et al., 2015). The selected miR395a-targeting PPR mRNA consists of both E and DYW domains, confirmed by the PPR database (https://ppr.plantenergy.uwa.edu.au/), which shows it could be involved in chloroplast RNA editing and processing. It was also revealed that the PPR40 protein acts as a mediator in the mitochondrial electron transport mechanism in Arabidopsis (Zsigmond et al., 2008). Pentatricopeptide repeat for germination on NaCl (PGN) was shown to be involved in the defense mechanism against necrotrophic fungi, *Botrytis cinerea,* in Arabidopsis (Laluk et al., 2011). Interestingly, the same study also reported that overexpression of the PGN resulted in the susceptibility toward fungal infection (Laluk et al., 2011). This suggested that PPR overexpression could lead to over-editing and misprocessing of chloroplast and mitochondrial RNA that might lead to its disruption. From the current study, it was revealed that the upregulation of PPR is a target gene of miR-395a and shows downregulation upon *R. solani* infection. In correlation with the previous observation from Laluk et al., 2011, *R. solani* infection leads to increased expression of PPR via miR-395a which could promote organellar necrosis by over-editing and misprocessing of their RNAs. However, further studies are warranted to decipher such mechanisms.

Zma-miR408b is a homolog of Osa-miR408-3p and was reported to target the genes such as *Zm00001d031257* (encoding cupredoxin), *Zm00001d028797* (encoding laccase 13), and *Zm00001d010887* (encoding serine/threonine-protein phosphatase BSL1) which are involved in the disease-resistant mechanism (Zhou et al., 2020). Subsequently, they demonstrated that the augmented expression of Zma-miR408b led to reduction in the disease resistance against *Fusarium verticillioides*. In correlation with the aforementioned observation, we found that Osa-miR408-3p decreased levels in *R. solani*-infected rice plants suggesting plants might be activating the resistance mechanisms. However, such presumptions should be determined in the future.

The miR171 family is reported as one of the most conserved miRNA families in the plant system (Axtell and Bowman, 2008). In rice, the miR171 family consists of a total of 14 members with the mixture of evolutionarily conserved and recently emerged miRNAs. Osa-miR171i is the member of the recently emerged miRNAs due to the presence of additional mutation events compared to other conserved miRNAs (Zhu et al., 2015). From the BLAST sequence alignment, miRBase shows that Osa-miR171i is specific to the rice species. We also searched in other newly emerged miRNA databases such as PMRD (plant microRNA database) (Zhang et al., 2010) and PmiREN (Plant miRNA ENcyclopedia) (Guo et al., 2020) to find the Osa-miR171i equivalents in other plant species. Although we are unable to find any in PMRD, but found one in PmiREN with two mismatches in *Citrus sinensis* (sweet orange group) by aligning to Csi-miR170a. To the best of our knowledge, till now there are no experimental reports on expression studies of either Osa-miR171i-5p or its targets. In a recent study of rice small RNA-sequencing upon *M. oryzae* infection, Osa-miR171i-5p shows >2-fold upregulation at 24 hpi and >1.5-fold upregulation at 48 and 120 hpi (Zhang et al., 2018). Similarly, the current study shows 1.5- to 2-fold upregulation at 60 and 72 hpi of *R. solani* post-infection and a slight downregulation at 24 hpi. These observations reveal that Osa-miR171i-5p could play a specific role in necrotrophic fungal diseases in rice.

Osa-miR1861d encodes from the clustered miRNA gene known as polycistronic transcript. Osa-miR1861d lies on chromosome 4 across rice species except for *Oryza nivara* (AA) in which it is located on chromosome 1, and the other miRNA clustered with this is miR 1861e (Baldrich et al., 2016). The other polycistron of the 1861 family, Osa-miR1861b-1861c, acts as a positive regulator for the rice immunity against *M. oryzae* (Salvador Guirao, 2018). Several other reports have shown the expression of members of the miR1861 family such as miR 1861a, miR 1861i, and miR 1861h during fungal infections like *M. oryzae* and *R. solani* in rice (Li et al., 2016; Wenlei et al., 2020; Arora et al., 2021). The miRNAs encoded by the clustered miRNA gene are involved in the regulation of the diverse functional gene expression responsible for plant development and stress conditions (Baldrich et al., 2016). Osa-miR1861 displayed a relatively high expression during grain filling suggesting that they may be important regulators of rice grain development (Yi et al., 2013; Hu et al., 2018). Some evidence proved that miRNAs are involved in carbohydrate and nitrogen metabolism. For example, Osa-miR1861, whose target mRNAs encode a starch-binding domain-containing protein (*Os01g63810*), is involved in starch degradation (Yi et al., 2013).

In the current study, we have performed the time series qRT-PCR for the selected miRNA and its target mRNAs. The quantification analysis shows the inverse correlation of miR395a, miR408-3p, and miR171i-5p with its target mRNA PPR, DNA-3-methyladenine glycosylase I, and BRO1, respectively. The other miRNAs and their target mRNAs show an incoherent correlation of quantification; this could be due to the spatial and temporal expression of miRNAs and mRNAs (Wang et al., 2019), isoform expression (Chaudhary et al., 2019), miRNA sponging ability by circRNAs, and long-noncoding RNAs (Guria et al., 2020).

## Conclusion

The present study shows the expression dynamics of several rice miRNAs upon *R. solani* infection. Our study reported the experimental validation by qRT-PCR and 5’ RLM-RACE and presented the top 5 miRNA expression levels and its mRNA target validation. Further gene transformation studies and *in vivo* and *in planta* validation of the identified miRNAs will dissect its role during pathogen infection. Other experimental studies such as overexpression and downregulation of the miRNA gene could provide the pathogen-associated gene network.

## Data Availability

The datasets presented in this study can be found in online repositories. The names of the repository/repositories and accession number(s) can be found in the article/Supplementary Material.

## Supplementary Material

Supplementary Figure S1 Heat map and MA-plots. Heat map and MA-plots were generated between control and infected samples from 12 hpi, 36 hpi and 48 hpi. Yellow-low-level expression; Red-high-level expression. The red dots in MA-plots represents the potential miRNAs expressed upon *R. solani* infection.

Supplementary Figure S2 RT-PCR of miRNAs. **(A,B)** Expected band was obtained in RT-PCR with each of the four miRNAs, namely, miR408-3P, miR395a, miR171i-3p and miR1861d. Low range ladder (25–700 bp) (Gene ruler, Thermo scientific) was used as reference marker along with 5.8s (positive control) and water control.

Supplementary Figure S3 Semi-quantitative PCR for miRNA. Semi-quantitative PCR was carried out for miR395a and miR408-3p using specific primers for uninfected and infected samples at different time points post *R. solani* infection along with negative (water, RNA) and positive control (5.8s rRNA). Low range ladder (25–700 bp) (Gene ruler, Thermo scientific) was used as reference marker.

Supplementary Figure S4 Semi-quantitative PCR of miRNA target gene. Semi-quantitative PCR was carried out for DNA-3-methyladenine glycosylase I, Carbon catabolite repressor (CCR4b), HUA enhancer 2, Argonaute2 and Pentatricopeptide specific genes for uninfected and infected samples at different time points post *R. solani* infection along with negative (water, RNA) and positive controls (5.8s and/or actin). 1Kb plus ladder (75–20,000 bp) (Gene ruler, Thermo scientific) was used as reference marker.

Supplementary Figure S5
**(A)** Restriction digestion of RACE Inner PCR product. The 5’RACE inner PCR product for Pentatricopeptide was digested with the MseI and the expected product size of 84 bp and 77 bp were observed (Digested) when compared with undigested product (Undigested). Few undigested products were also observed along with the digested product due to less efficacy of enzyme or possibility of having non-specific products. Low range ladder (25–700 bp) (Gene ruler, Thermo scientific) was used as reference marker. **(B)** Sequencing data of the Pentatricopeptide RACE-PCR clone. The yellow background sequence is the expected Pentatricopeptide and the vector backbone sequence was highlighted with grey background.

Supplementary Figure S6
**(A)** Restriction digestion of pGEMT-Easy vector cloned pre-NmiR1 PCR product. The clone was digested with both the *EcoRI* and *SspI*. The expected product size of 115 bp and 2992 bp were observed when compared with undigested product of *EcoRI*. For *SspI* digestion, the expected product size of 182 bp, 848 bp and 2095 bp were observed when compared with undigested product. 1 kb plus ladder (75–20,000 bp) (Gene ruler, Thermo scientific) was used as reference marker. **(B)** Sequencing data of the pre-NmiR1 cloned pGEMT-Easy vector. The yellow background sequence is the expected pre-NmiR1 sequence and the vector backbone sequence was highlighted with grey background.

Supplementary Table S1 List of primers used in the study.

Supplementary Table S2 Table showing analysis of NGS reads for miRNA identification from 18 sequencing libraries.

Supplementary Table S3 Expression of top upregulated and downregulated rice miRNAs at different time points post *R. solani* infection.

Supplementary Table S4 Table showing number of supporting reads for the precursor novel miRNA, NmiR1, identified using Mireap at different time points.
