# Supplementary material for: An Insight Into Pentatricopeptide-Mediated Chloroplast Necrosis via microRNA395a During Rhizoctonia solani Infection
Source: Front Genet. 2022 May 30;13:869465. doi: 10.3389/fgene.2022.869465 (PMC9189367; doi:10.3389/fgene.2022.869465)
Supplement: Supplementary file 1 [file Presentation1.pptx]

## Slide 1
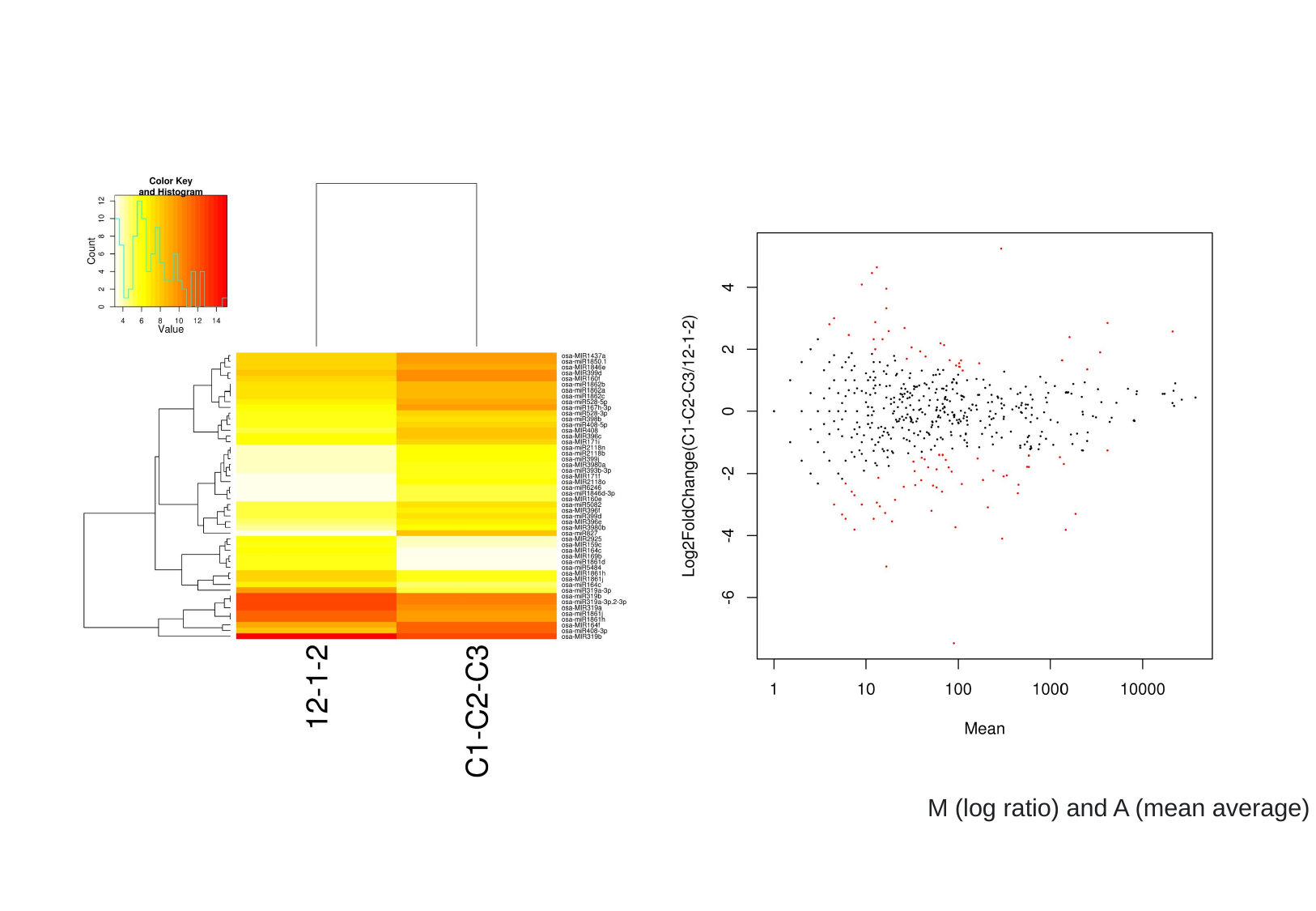

M (log ratio) and A (mean average)

## Slide 2
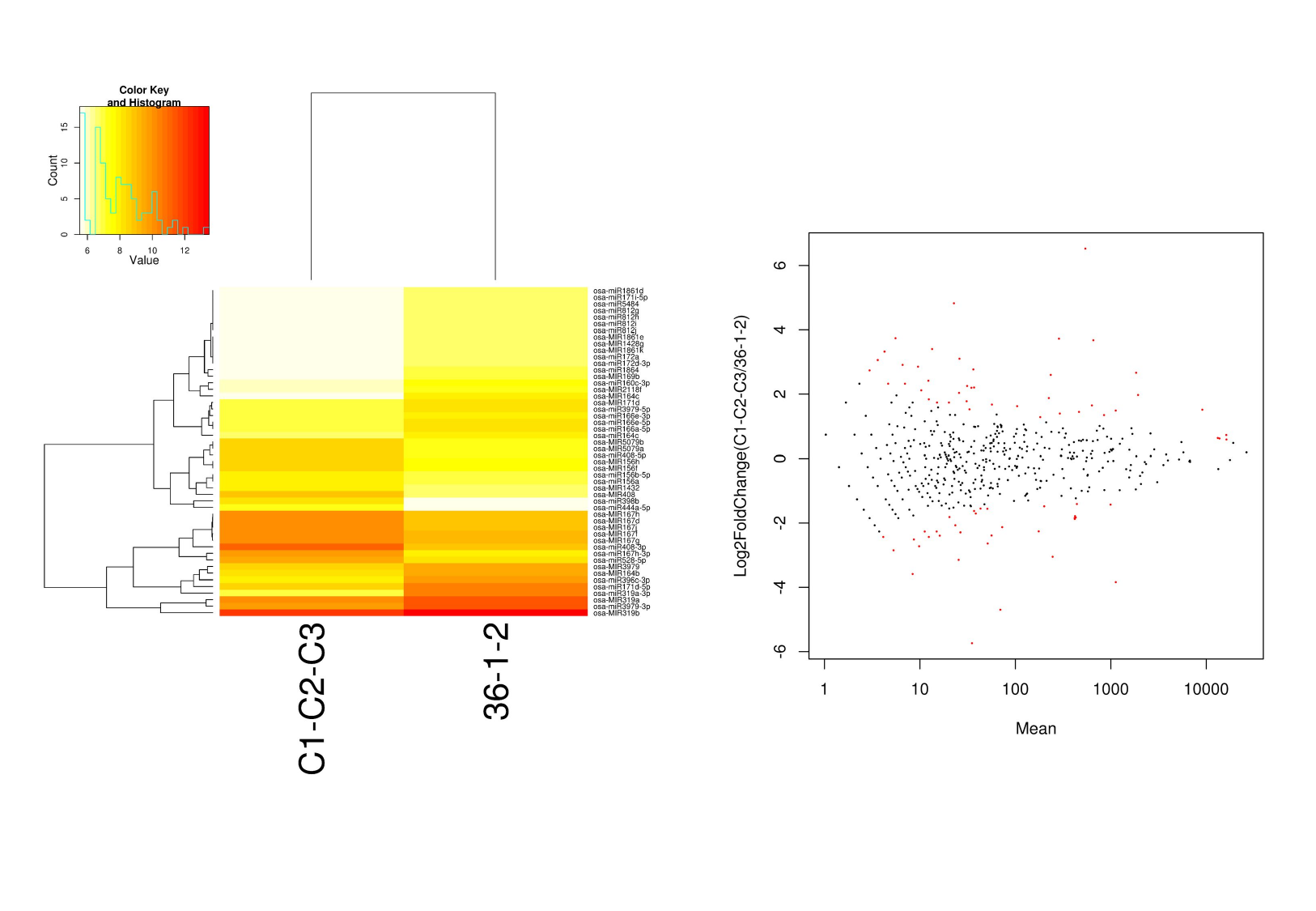

## Slide 3
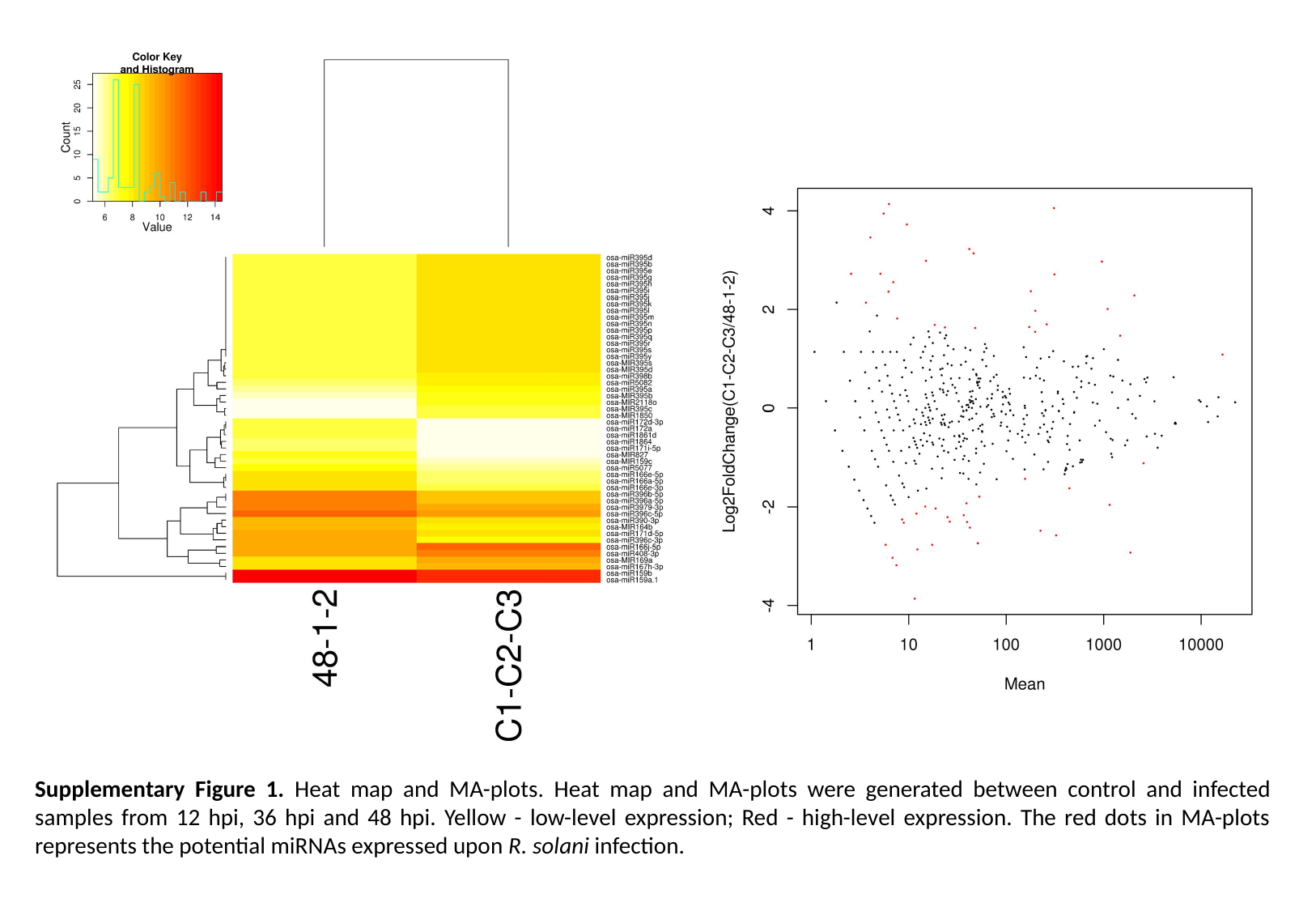

Supplementary Figure 1. Heat map and MA-plots. Heat map and MA-plots were generated between control and infected samples from 12 hpi, 36 hpi and 48 hpi. Yellow - low-level expression; Red - high-level expression. The red dots in MA-plots represents the potential miRNAs expressed upon R. solani infection.
