# Supplementary material for: An Insight Into Pentatricopeptide-Mediated Chloroplast Necrosis via microRNA395a During Rhizoctonia solani Infection
Source: Front Genet. 2022 May 30;13:869465. doi: 10.3389/fgene.2022.869465 (PMC9189367; doi:10.3389/fgene.2022.869465)
Supplement: Supplementary file 3 [file Presentation4.pptx]

## Slide 1
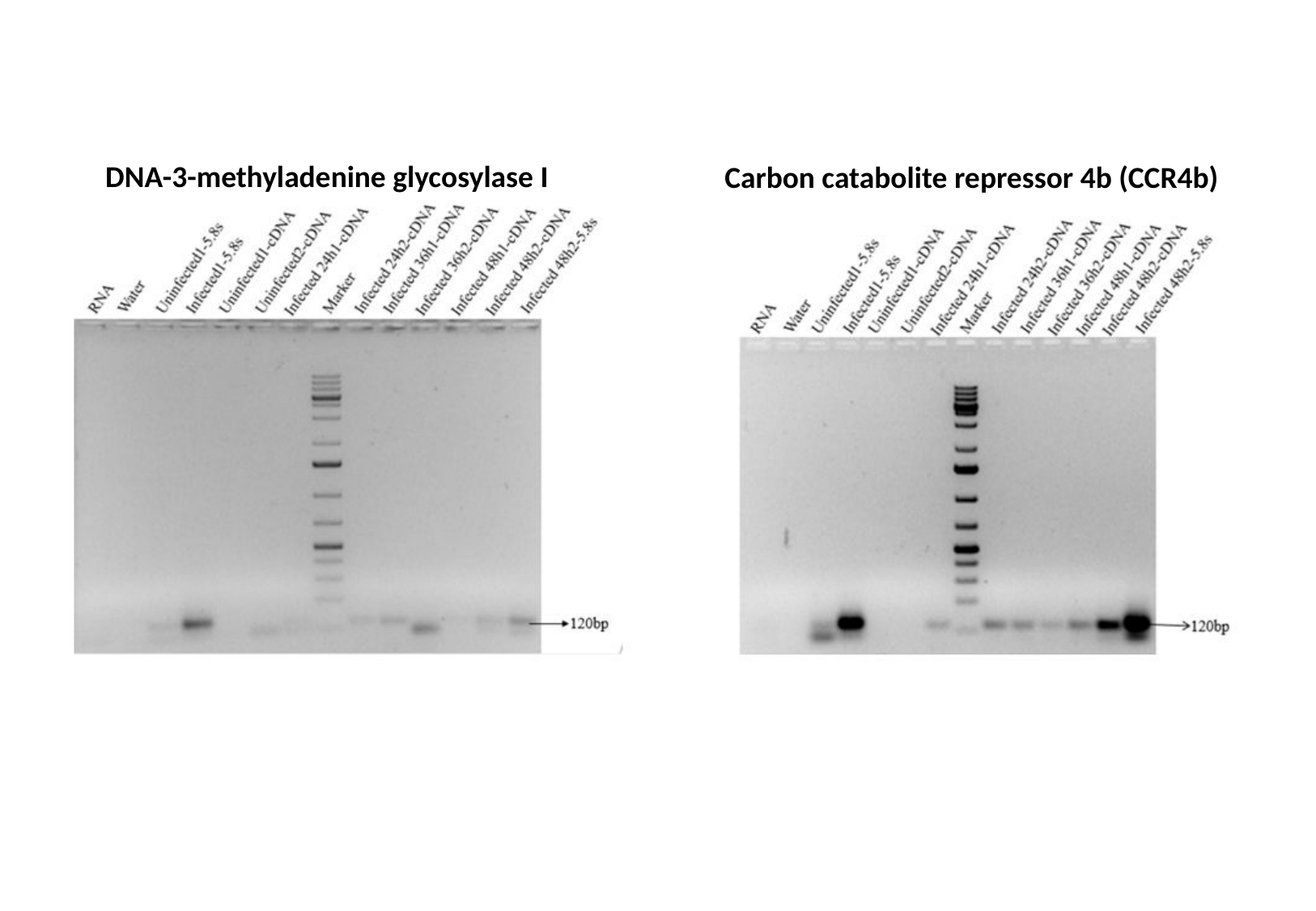

DNA-3-methyladenine glycosylase I
Carbon catabolite repressor 4b (CCR4b)

## Slide 2
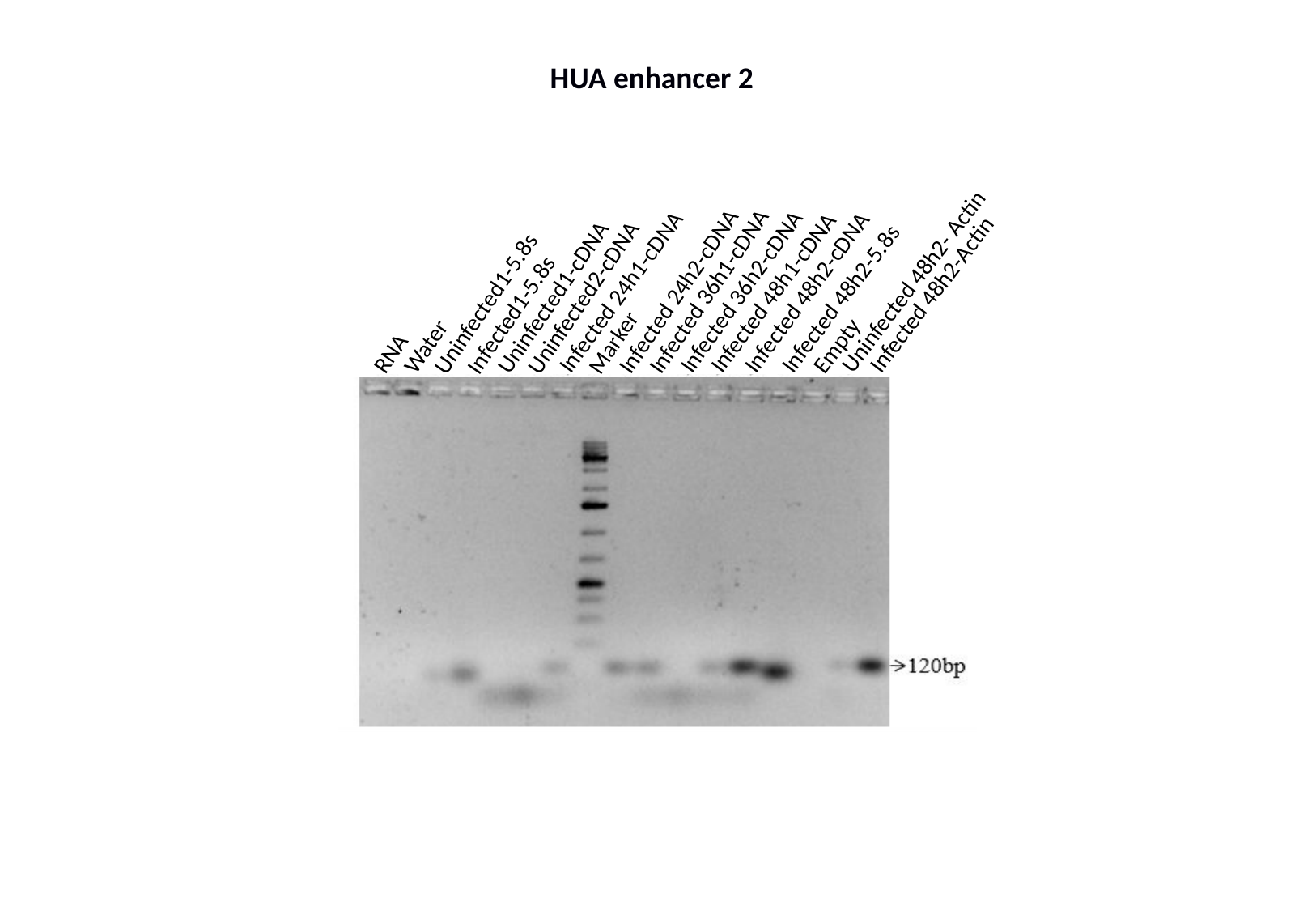

HUA enhancer 2
Uninfected2-cDNA
Uninfected1-cDNA
Infected 24h2-cDNA
Infected 36h1-cDNA
Uninfected 48h2- Actin
Uninfected1-5.8s
Infected1-5.8s
Infected 36h2-cDNA
Infected 24h1-cDNA
Infected 48h2-cDNA
Infected 48h1-cDNA
Infected 48h2-Actin
Infected 48h2-5.8s
RNA
Water
Marker
Empty

## Slide 3
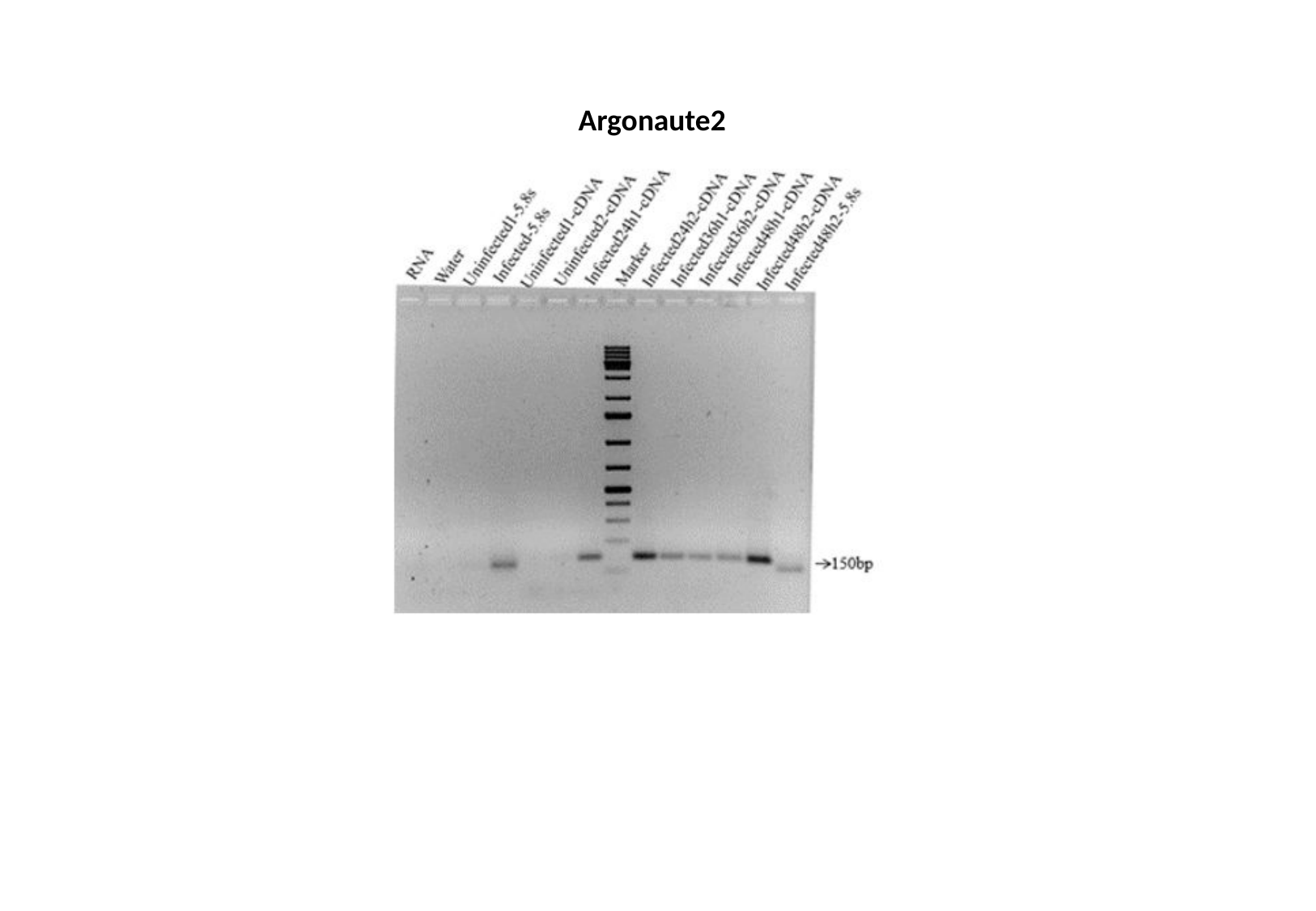

Argonaute2

## Slide 4
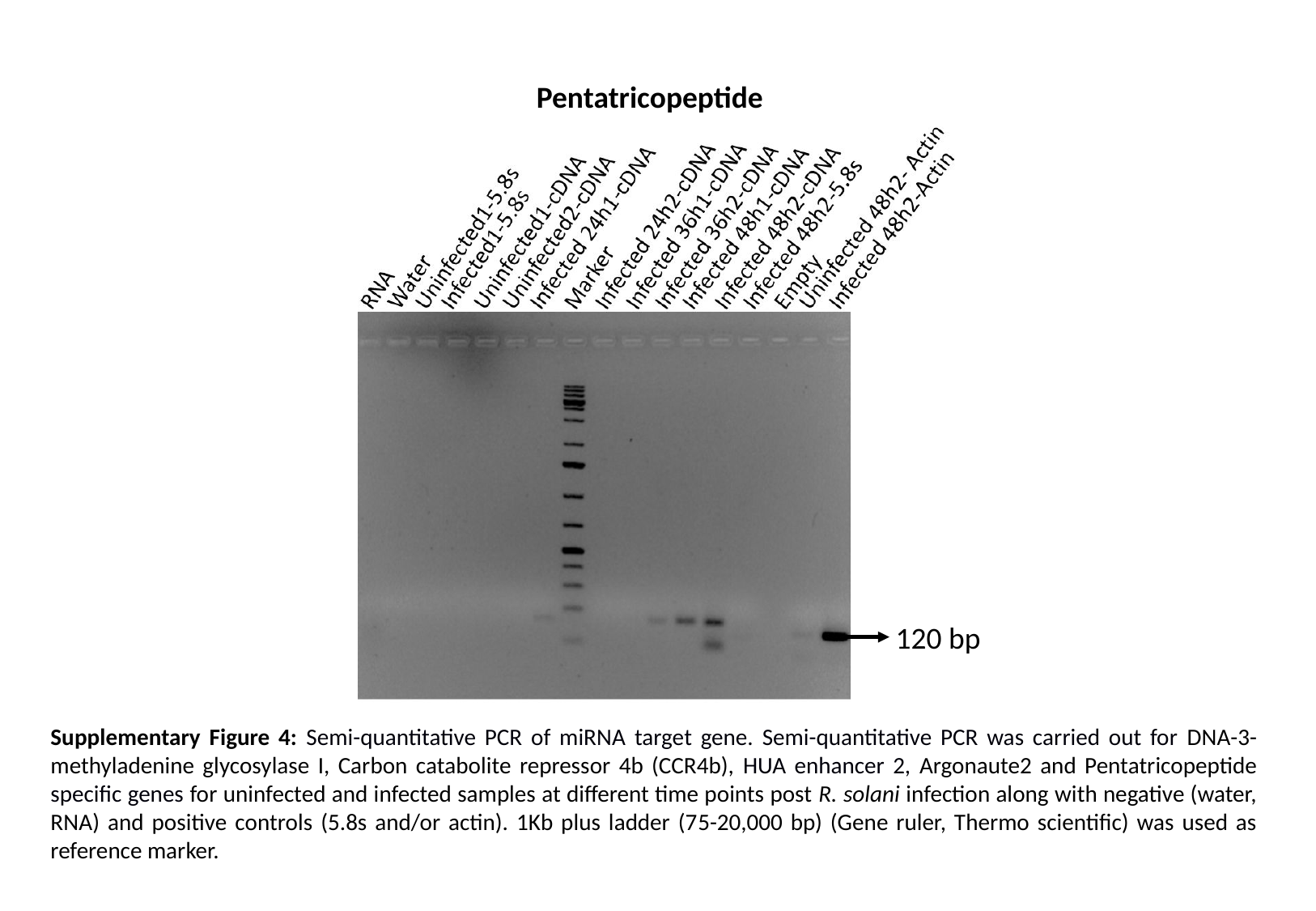

Pentatricopeptide
120 bp
Supplementary Figure 4: Semi-quantitative PCR of miRNA target gene. Semi-quantitative PCR was carried out for DNA-3-methyladenine glycosylase I, Carbon catabolite repressor 4b (CCR4b), HUA enhancer 2, Argonaute2 and Pentatricopeptide specific genes for uninfected and infected samples at different time points post R. solani infection along with negative (water, RNA) and positive controls (5.8s and/or actin). 1Kb plus ladder (75-20,000 bp) (Gene ruler, Thermo scientific) was used as reference marker.
