# Supplementary material for: An Insight Into Pentatricopeptide-Mediated Chloroplast Necrosis via microRNA395a During Rhizoctonia solani Infection
Source: Front Genet. 2022 May 30;13:869465. doi: 10.3389/fgene.2022.869465 (PMC9189367; doi:10.3389/fgene.2022.869465)
Supplement: Supplementary file 4 [file Presentation6.pptx]

## Slide 1
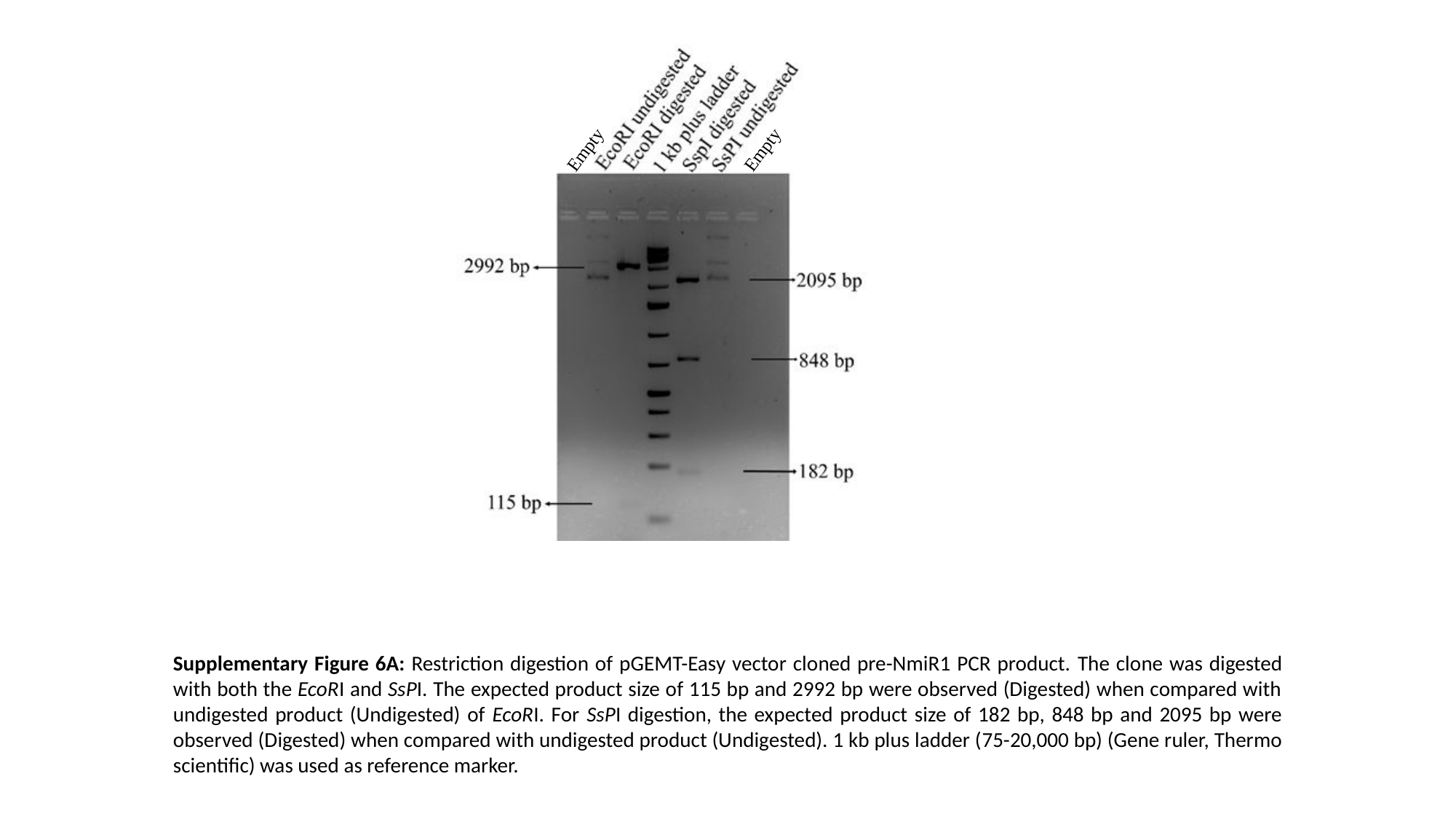

Empty
Empty
Supplementary Figure 6A: Restriction digestion of pGEMT-Easy vector cloned pre-NmiR1 PCR product. The clone was digested with both the EcoRI and SsPI. The expected product size of 115 bp and 2992 bp were observed (Digested) when compared with undigested product (Undigested) of EcoRI. For SsPI digestion, the expected product size of 182 bp, 848 bp and 2095 bp were observed (Digested) when compared with undigested product (Undigested). 1 kb plus ladder (75-20,000 bp) (Gene ruler, Thermo scientific) was used as reference marker.

## Slide 2
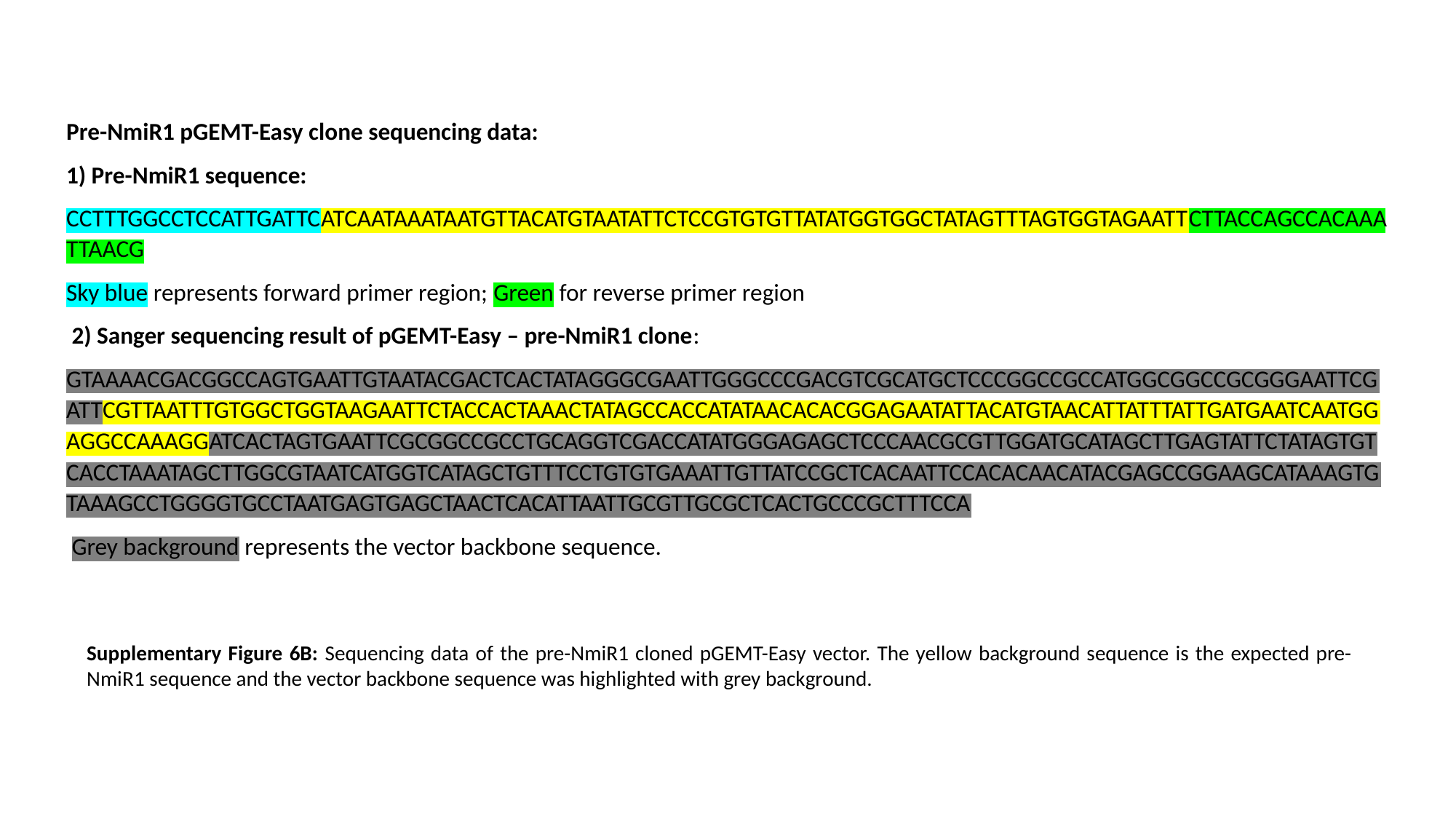

Pre-NmiR1 pGEMT-Easy clone sequencing data:
1) Pre-NmiR1 sequence:
CCTTTGGCCTCCATTGATTCATCAATAAATAATGTTACATGTAATATTCTCCGTGTGTTATATGGTGGCTATAGTTTAGTGGTAGAATTCTTACCAGCCACAAATTAACG
Sky blue represents forward primer region; Green for reverse primer region
 2) Sanger sequencing result of pGEMT-Easy – pre-NmiR1 clone:
GTAAAACGACGGCCAGTGAATTGTAATACGACTCACTATAGGGCGAATTGGGCCCGACGTCGCATGCTCCCGGCCGCCATGGCGGCCGCGGGAATTCGATTCGTTAATTTGTGGCTGGTAAGAATTCTACCACTAAACTATAGCCACCATATAACACACGGAGAATATTACATGTAACATTATTTATTGATGAATCAATGGAGGCCAAAGGATCACTAGTGAATTCGCGGCCGCCTGCAGGTCGACCATATGGGAGAGCTCCCAACGCGTTGGATGCATAGCTTGAGTATTCTATAGTGTCACCTAAATAGCTTGGCGTAATCATGGTCATAGCTGTTTCCTGTGTGAAATTGTTATCCGCTCACAATTCCACACAACATACGAGCCGGAAGCATAAAGTGTAAAGCCTGGGGTGCCTAATGAGTGAGCTAACTCACATTAATTGCGTTGCGCTCACTGCCCGCTTTCCA
 Grey background represents the vector backbone sequence.
Supplementary Figure 6B: Sequencing data of the pre-NmiR1 cloned pGEMT-Easy vector. The yellow background sequence is the expected pre-NmiR1 sequence and the vector backbone sequence was highlighted with grey background.
