# Supplementary material for: An Insight Into Pentatricopeptide-Mediated Chloroplast Necrosis via microRNA395a During Rhizoctonia solani Infection
Source: Front Genet. 2022 May 30;13:869465. doi: 10.3389/fgene.2022.869465 (PMC9189367; doi:10.3389/fgene.2022.869465)
Supplement: Supplementary file 6 [file Presentation3.pptx]

## Slide 1
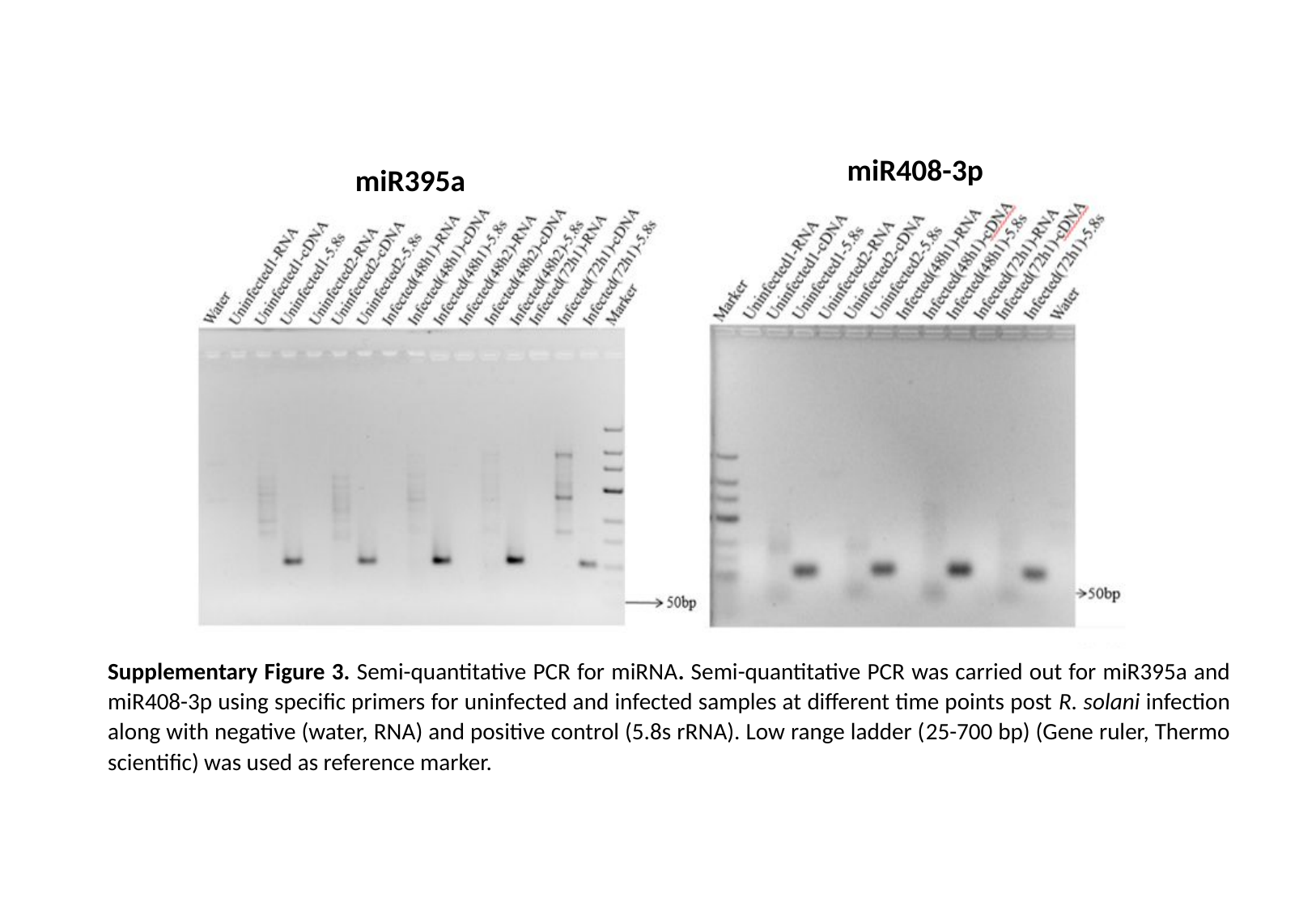

miR408-3p
miR395a
Supplementary Figure 3. Semi-quantitative PCR for miRNA. Semi-quantitative PCR was carried out for miR395a and miR408-3p using specific primers for uninfected and infected samples at different time points post R. solani infection along with negative (water, RNA) and positive control (5.8s rRNA). Low range ladder (25-700 bp) (Gene ruler, Thermo scientific) was used as reference marker.
