# Supplementary material for: An Insight Into Pentatricopeptide-Mediated Chloroplast Necrosis via microRNA395a During Rhizoctonia solani Infection
Source: Front Genet. 2022 May 30;13:869465. doi: 10.3389/fgene.2022.869465 (PMC9189367; doi:10.3389/fgene.2022.869465)
Supplement: Supplementary file 7 [file Presentation2.pptx]

## Slide 1
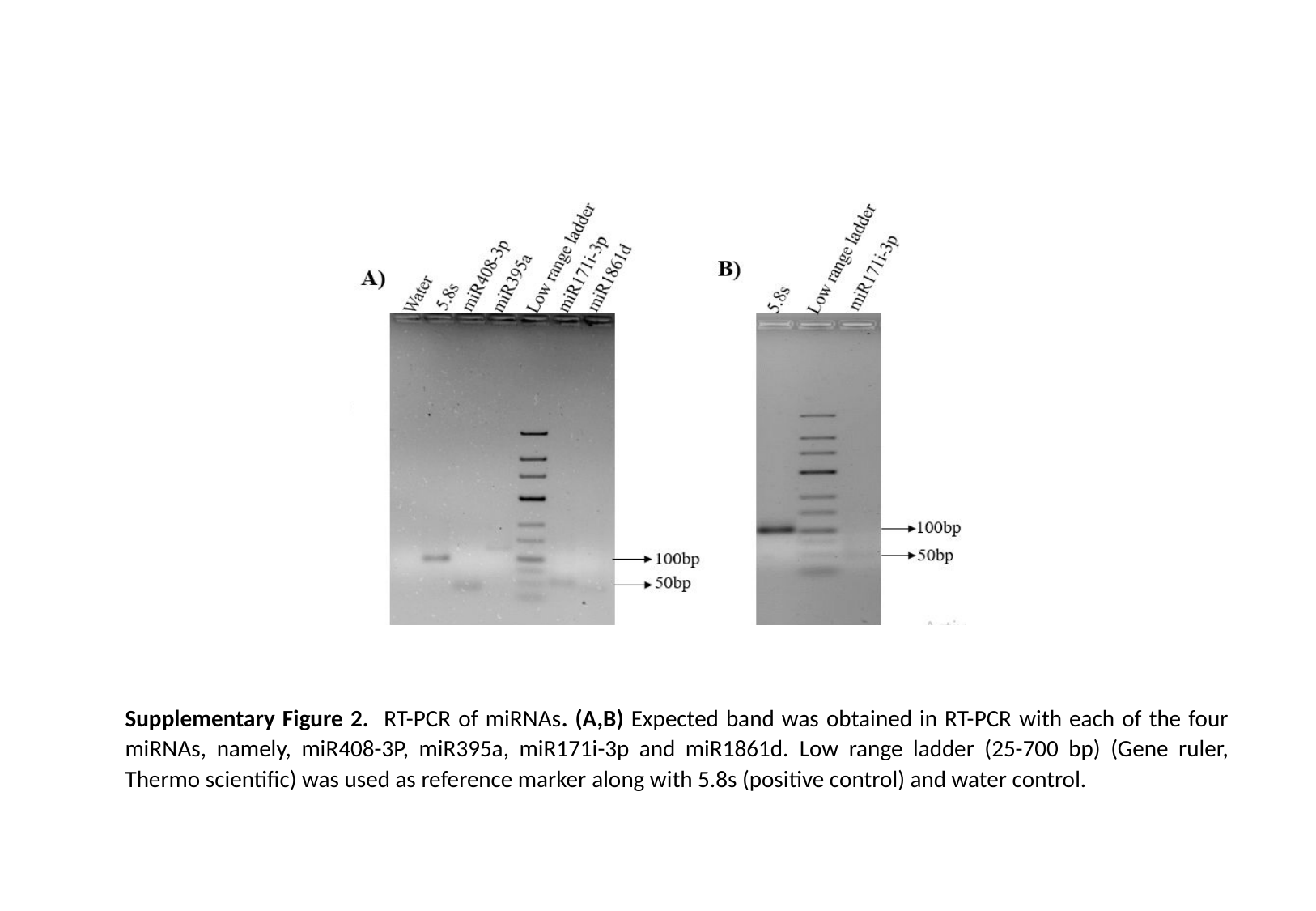

Supplementary Figure 2. RT-PCR of miRNAs. (A,B) Expected band was obtained in RT-PCR with each of the four miRNAs, namely, miR408-3P, miR395a, miR171i-3p and miR1861d. Low range ladder (25-700 bp) (Gene ruler, Thermo scientific) was used as reference marker along with 5.8s (positive control) and water control.
