# Supplementary material for: An Insight Into Pentatricopeptide-Mediated Chloroplast Necrosis via microRNA395a During Rhizoctonia solani Infection
Source: Front Genet. 2022 May 30;13:869465. doi: 10.3389/fgene.2022.869465 (PMC9189367; doi:10.3389/fgene.2022.869465)
Supplement: Supplementary file 9 [file Presentation5.pptx]

## Slide 1
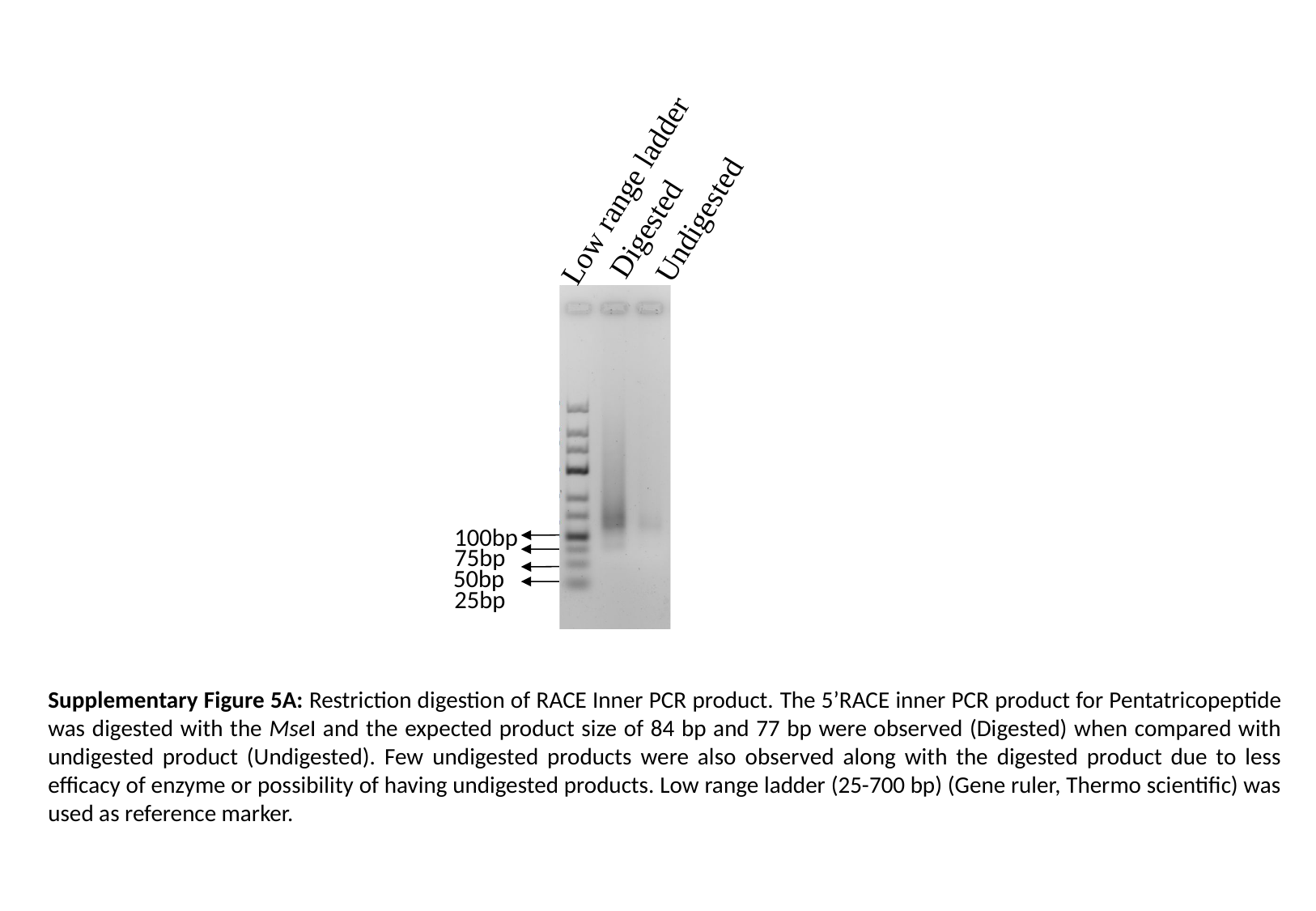

Low range ladder
Undigested
Digested
100bp
75bp
50bp
25bp
Supplementary Figure 5A: Restriction digestion of RACE Inner PCR product. The 5’RACE inner PCR product for Pentatricopeptide was digested with the MseI and the expected product size of 84 bp and 77 bp were observed (Digested) when compared with undigested product (Undigested). Few undigested products were also observed along with the digested product due to less efficacy of enzyme or possibility of having undigested products. Low range ladder (25-700 bp) (Gene ruler, Thermo scientific) was used as reference marker.

## Slide 2
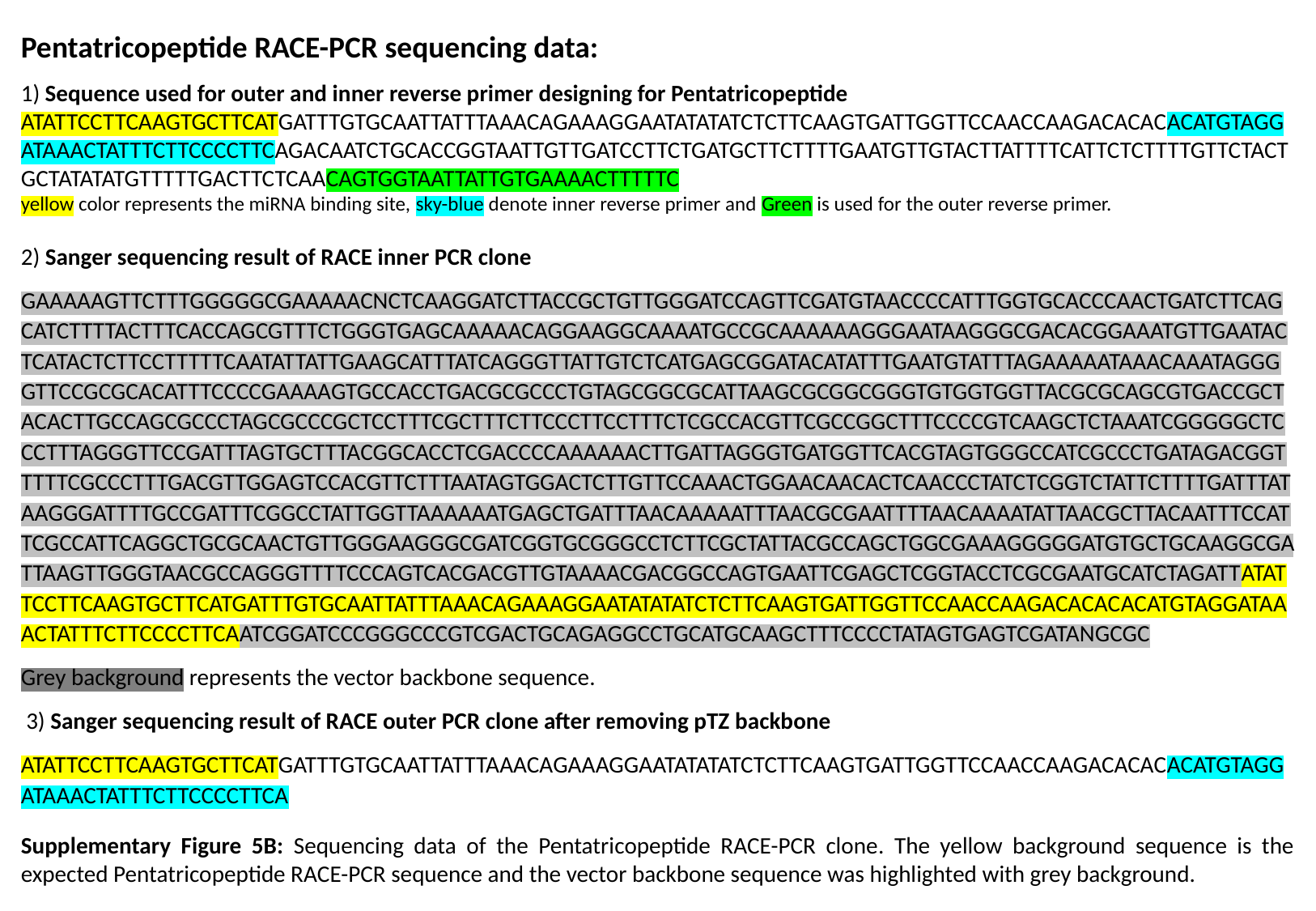

Pentatricopeptide RACE-PCR sequencing data:
1) Sequence used for outer and inner reverse primer designing for Pentatricopeptide
ATATTCCTTCAAGTGCTTCATGATTTGTGCAATTATTTAAACAGAAAGGAATATATATCTCTTCAAGTGATTGGTTCCAACCAAGACACACACATGTAGGATAAACTATTTCTTCCCCTTCAGACAATCTGCACCGGTAATTGTTGATCCTTCTGATGCTTCTTTTGAATGTTGTACTTATTTTCATTCTCTTTTGTTCTACTGCTATATATGTTTTTGACTTCTCAACAGTGGTAATTATTGTGAAAACTTTTTC
yellow color represents the miRNA binding site, sky-blue denote inner reverse primer and Green is used for the outer reverse primer.
2) Sanger sequencing result of RACE inner PCR clone
GAAAAAGTTCTTTGGGGGCGAAAAACNCTCAAGGATCTTACCGCTGTTGGGATCCAGTTCGATGTAACCCCATTTGGTGCACCCAACTGATCTTCAGCATCTTTTACTTTCACCAGCGTTTCTGGGTGAGCAAAAACAGGAAGGCAAAATGCCGCAAAAAAGGGAATAAGGGCGACACGGAAATGTTGAATACTCATACTCTTCCTTTTTCAATATTATTGAAGCATTTATCAGGGTTATTGTCTCATGAGCGGATACATATTTGAATGTATTTAGAAAAATAAACAAATAGGGGTTCCGCGCACATTTCCCCGAAAAGTGCCACCTGACGCGCCCTGTAGCGGCGCATTAAGCGCGGCGGGTGTGGTGGTTACGCGCAGCGTGACCGCTACACTTGCCAGCGCCCTAGCGCCCGCTCCTTTCGCTTTCTTCCCTTCCTTTCTCGCCACGTTCGCCGGCTTTCCCCGTCAAGCTCTAAATCGGGGGCTCCCTTTAGGGTTCCGATTTAGTGCTTTACGGCACCTCGACCCCAAAAAACTTGATTAGGGTGATGGTTCACGTAGTGGGCCATCGCCCTGATAGACGGTTTTTCGCCCTTTGACGTTGGAGTCCACGTTCTTTAATAGTGGACTCTTGTTCCAAACTGGAACAACACTCAACCCTATCTCGGTCTATTCTTTTGATTTATAAGGGATTTTGCCGATTTCGGCCTATTGGTTAAAAAATGAGCTGATTTAACAAAAATTTAACGCGAATTTTAACAAAATATTAACGCTTACAATTTCCATTCGCCATTCAGGCTGCGCAACTGTTGGGAAGGGCGATCGGTGCGGGCCTCTTCGCTATTACGCCAGCTGGCGAAAGGGGGATGTGCTGCAAGGCGATTAAGTTGGGTAACGCCAGGGTTTTCCCAGTCACGACGTTGTAAAACGACGGCCAGTGAATTCGAGCTCGGTACCTCGCGAATGCATCTAGATTATATTCCTTCAAGTGCTTCATGATTTGTGCAATTATTTAAACAGAAAGGAATATATATCTCTTCAAGTGATTGGTTCCAACCAAGACACACACATGTAGGATAAACTATTTCTTCCCCTTCAATCGGATCCCGGGCCCGTCGACTGCAGAGGCCTGCATGCAAGCTTTCCCCTATAGTGAGTCGATANGCGC
Grey background represents the vector backbone sequence.
 3) Sanger sequencing result of RACE outer PCR clone after removing pTZ backbone
ATATTCCTTCAAGTGCTTCATGATTTGTGCAATTATTTAAACAGAAAGGAATATATATCTCTTCAAGTGATTGGTTCCAACCAAGACACACACATGTAGGATAAACTATTTCTTCCCCTTCA
Supplementary Figure 5B: Sequencing data of the Pentatricopeptide RACE-PCR clone. The yellow background sequence is the expected Pentatricopeptide RACE-PCR sequence and the vector backbone sequence was highlighted with grey background.
